# Human milk oligosaccharides protect against the development of autoimmune diabetes in NOD-mice

**DOI:** 10.1038/s41598-018-22052-y

**Published:** 2018-03-01

**Authors:** Ling Xiao, Belinda van’t Land, Phillip A. Engen, Ankur Naqib, Stefan J. Green, Angie Nato, Thea Leusink-Muis, Johan Garssen, Ali Keshavarzian, Bernd Stahl, Gert Folkerts

**Affiliations:** 1 Utrecht University, Faculty of Science, Department of Pharmaceutical Sciences, Division of Pharmacology, Utrecht, The Netherlands; 2 Nutricia Research, Department of Immunology/Human milk research platform, Utrecht, The Netherlands; 3 University Medical Center Utrecht, The Wilhelmina Children’s Hospital, Laboratory of Translational Immunology, Utrecht, The Netherlands; 4 Department of Internal Medicine, Division of Digestive Diseases and Nutrition, Rush University Medical Center, Chicago, IL USA; 5 DNA Services Facility, Research Resources Center, Department of Biological Sciences, University of Illinois at Chicago, Chicago, IL USA; 6 Department of Pharmacology, Department of Physiology, Rush University Medical Center, Chicago, IL USA

## Abstract

Development of Type 1 diabetes (T1D) is influenced by non-genetic factors, such as optimal microbiome development during early life that “programs” the immune system. Exclusive and prolonged breastfeeding is an independent protective factor against the development of T1D, likely via bioactive components. Human Milk Oligosaccharides (HMOS) are microbiota modulators, known to regulate immune responses directly. Here we show that early life provision (only for a period of six weeks) of 1% authentic HMOS (consisting of both long-chain, as well as short-chain structures), delayed and suppressed T1D development in non-obese diabetic mice and reduced development of severe pancreatic insulitis in later life. These protective effects were associated with i) beneficial alterations in fecal microbiota composition, ii) anti-inflammatory microbiota-generating metabolite (i.e. short chain fatty acids (SCFAs)) changes in fecal, as well as cecum content, and iii) induction of anti-diabetogenic cytokine profiles. Moreover, *in vitro* HMOS combined with SCFAs induced development of tolerogenic dendritic cells (tDCs), priming of functional regulatory T cells, which support the protective effects detected *in vivo*. In conclusion, HMOS present in human milk are therefore thought to be vital in the protection of children at risk for T1D, supporting immune and gut microbiota development in early life.

## Introduction

Exclusive and prolonged breast-feeding have been identified as independent protective factor against insulin-dependent T1D development since 1984^1–3^. Aside from reducing risk factors such as early introduction of complementary foods including wheat proteins^3,4^, human milk *per se* might provide infants with anti-diabetic components, although to date these protective factors have not been identified. HMOS are a group of structurally diverse unconjugated short-chain, as well as long-chain glycans, found uniquely and abundantly (20–23 g/L in colostrum and 12–14 g/L in mature milk) in human milk^5^. The prebiotic effects of HMOS are well established (Reviewed by *Ling X*. *et al*.,^6^) and SCFAs derived from HMOS through microbial processing, influence innate as well as adaptive immunity. Microbial alterations influence the pancreatic immune environment^7^ (specific B cells and regulatory T cells (Tregs)^8^), leading to direct protection against the development of T1D in non-obese diabetic (NOD/ShiLtJ, a model of spontaneous diabetes development)^7,8^. Moreover, specific oligosaccharides in human milk are capable of shaping infants innate immune system through binding to DC-SIGN^9^, suggesting direct microbiota-independent immunomodulatory properties of HMOS. Collectively, these data indicate that HMOS are essential in microbiota and immune development of neonates. However, little is known about the contribution of HMOS in the protective effect of human milk on the development of autoimmune diseases such as T1D.

T1D results from autoimmune destruction of insulin-producing β cells of the pancreas. This Th1 and/or Th17-mediated disease involves deregulation of CD8 + T-cell and innate immune cells such as dendritic cells (DCs)^10^. Through shared lymphocytes homing receptors between gut and pancreas^11^, mucosal immune development may directly affect the pancreatic β-cell. Consequently, gut microbiota development and modulation seems a promising target in prevention of T1D. Interaction between intestinal microbes and host’s innate immune system within T1D was first described by Wen Li^12^. Indeed, microbiota modulation by either early-life exposure to antibiotics^13^, probiotics^14,15^, or dietary intervention with gluten free diet prevented onset and/or progression of this disease^16^. Hence, early in life there is a critical period for establishment of an optimal gut microbiota, which ‘educates’ infant’s immune system and development. Based on known protective effects of breastfeeding against T1D, and prebiotic effects of HMOS, we hypothesized that HMOS may contribute to the reduction of T1D development in breastfed infants. Therefore, the effect of dietary supplementation of the authentic complex mixture of HMOS isolated from human milk consisting of both short-chain as well as long-chain oligosaccharides, for a short period early in life on the development of T1D later in life in NOD mouse was tested. Furthermore, within *in vitro* DC-T-cell model, the direct immunomodulatory effects of HMOS and/or HMOS in the presence of SCFAs were confirmed.

## Results

### Early Dietary HMOS reduced diabetes incidence and pancreatic insulitis in NOD-mice later in life

**Figure 1:**
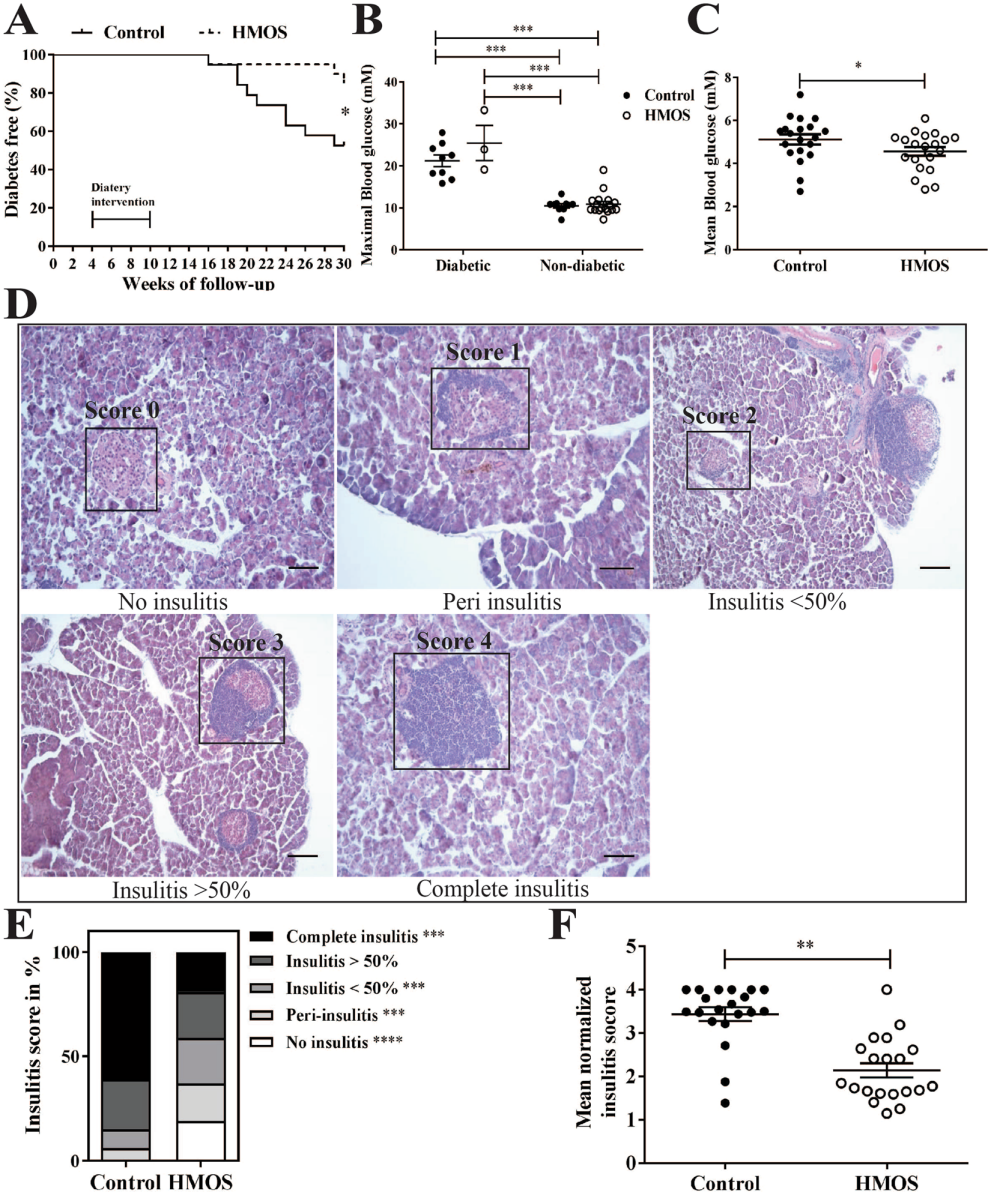
Early HMOS dietary intervention protects NOD-mice from onset and development of T1D. (**A**) Diabetes Free percentage in control (solid line trace, n = 19) and HMOS (dash line trace, n = 20) group over time. (**B**) Maximal measured blood glucose levels (mM) in control (black dots, n = 9 for diabetic and n = 11 for non-diabetic) and HMOS group (white dots, n = 3 for diabetic and n = 17 for non-diabetic) were grouped according to diabetic or non-diabetic status. (**C**) Mean Blood glucose (mM) of control (black dots, n = 19) and HMOS group (white dots, n = 20). (**D**) Degree of insulitis as scored on representative HE stained islets (range 0–4). Scale bars: 50 μm. (**E**) Average percentage of each score in the total islets counted in the Control or HMOS group. Average of 46 islets of each mouse from Control and HMOS mice were assigned insulitis scores. White bars present No-insulitis, light grey bars present Peri-insulitis, grey bars presents Insulitis <50%, dark grey bars present Insulitis >50%, and black bars present Complete-insulitis. (**F**) Mean value of mean normalized insulitis scores (range 0–4) of each mouse from control and HMOS groups are shown. Data are presented as mean ± SEM, n = 19–20/group. Statistical differences between groups are depicted as *p < 0.05, **p < 0.01, ***p < 0.001 and ****p < 0.0001 using Mann-Whitney U-test.

To determine the effects of HMOS on T1D development, four-week (Wk)-old female NOD-mice were provided with (or without) 1% HMOS containing diet from Wk4 until Wk10 of life. Clinical onset of diabetes was determined based on weekly monitored urine glucose levels. NOD-mice receiving HMOS showed delayed onset in disease development (22 ± 1.4 and 25 ± 4.5 weeks (mean ± SEM) for HMOS and control group respectively). The time of each diabetic mouse occurrence in both control and HMOS diet receiving groups were shown Fig. S1. At the end of the study, a significant reduction in the incidence of T1D was detected within NOD-mice receiving HMOS early in life (15% and 47.4% for HMOS and control group, p < 0.05 respectively, n = 19 for control, and n = 20 for HMOS group, Fig. 1A). Maximal measured blood glucose levels confirmed classification of non-diabetic and diabetic mice, since significantly higher levels were found between diabetic subgroups and non-diabetic subgroups (Fig. 1B). In addition, the mean blood glucose levels (mM) were significantly different between mouse from control and HMOS group (p < 0.05) (Fig. 1C). No changes between groups were observed in body weight over time (Fig. S1). Histological analysis of pancreatic islet inflammation (Degree of insulitis as scored on representative HE stained islets shown in Fig. 1D) revealed significantly (p < 0.001) reduced incidence of complete insulitis in HMOS-receiving NOD-mice (19%) compared to control NOD-mice (61%) (Fig. 1E). In mice receiving HMOS the mean normalized insulitis score was significantly lower (2.1 ± 0.16 (mean ± SEM) p < 0.001) than observed in control group (3.4 ± 0.16 (mean ± SEM)) (Fig. 1F). These data indicate that early HMOS supplementation can protect against T1D development.

### Dietary HMOS influenced cytokine profile in NOD-mice

**Figure 2:**
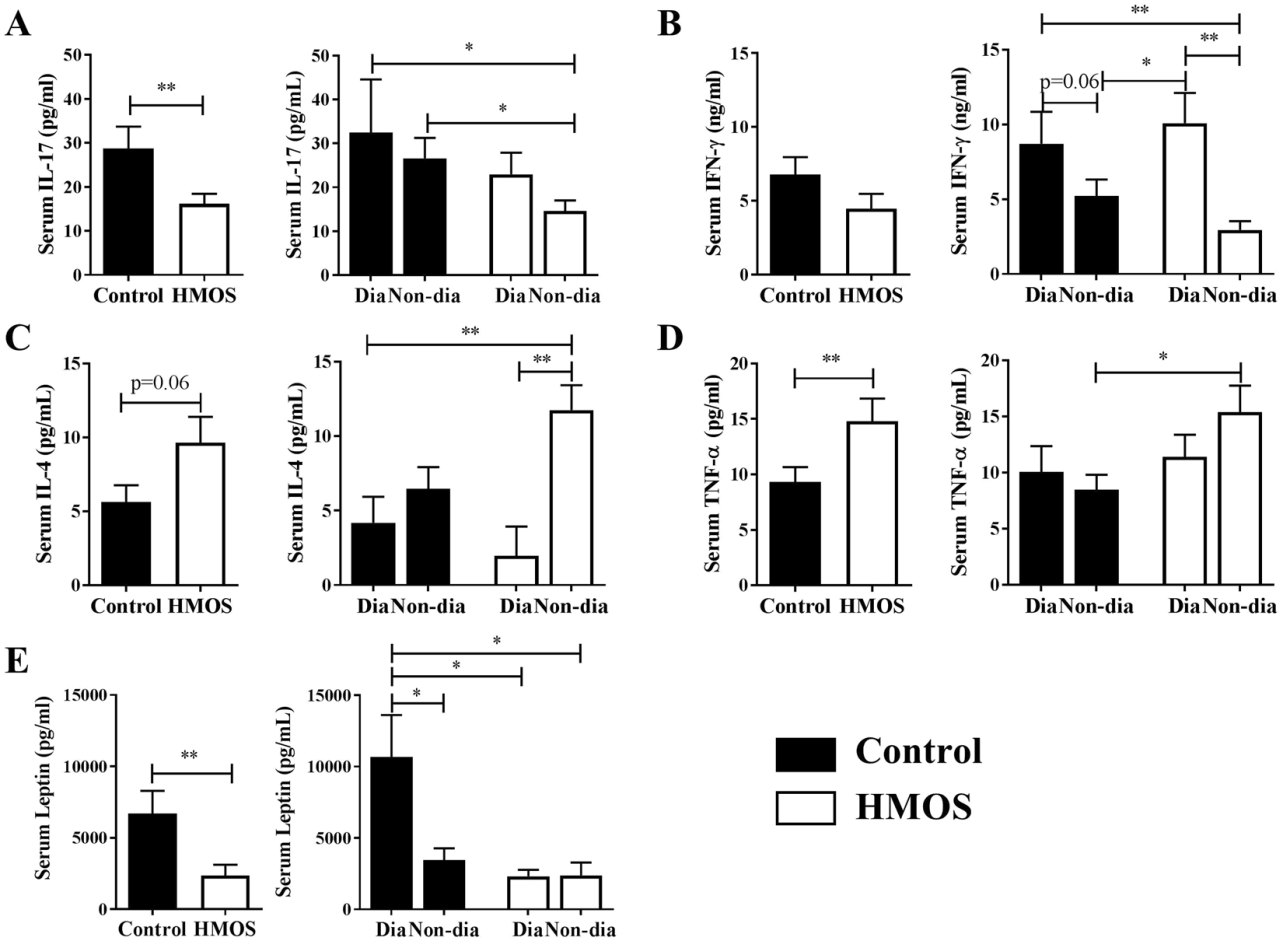
Early HMOS dietary intervention changes serum cytokines profile. The concentrations of (**A**) IL-17, (**B**) IFN-γ, (**C**) IL-4, (**D**) TNF-α, and (**E**) Leptin were analyzed in serum of HMOS-modulated and untreated NOD-micedepicted as either total group or separated as Diabetic (Dia) and Non-diabetic (Non-dia) mice as indicated. Data are presented as Mean ± SEM, n = 17/20/group. Statistical differences between groups are depicted as *p < 0.05 and **p < 0.01using Mann-Whitney U-test.

Both immune system regulation and microbiota modulation are suggested to impact T1D development^12^. To test the effects of dietary HMOS on systemic immunity, serum cytokines in NOD-mice were measured at endpoint. Significantly reduced concentrations of IL-17 were detected in HMOS-treated compared to control NOD-mice (p < 0.01, Fig. 2A), which was mainly present in the non-diabetic NOD-mice (p < 0.05, HMOS-Non-dia vs Control-Dia; p < 0.05, HMOS-Non-dia vs Control-Non-dia). Although no difference in the IFN-γ level between groups was found, non-diabetic mice from HMOS group showed significantly lower level than the two diabetic subgroups (p < 0.01, HMOS-Non-dia vs HMOS-Dia; p < 0.05, HMOS-Non-dia vs Control-Dia, Fig. 2B). In addition, a tendency towards an increased IL-4 level by HMOS dietary intervention was observed (p = 0.06, Fig. 2C), and significant difference was found between HMOS-Non-dia and HMOS-Dia (p < 0.01) subgroups, and HMOS-Non-dia and Control-Dia subgroups (p < 0.01). Surprisingly, TNF-α was significantly increased in HMOS-treated as compared to control NOD-mice (p < 0.01, Fig. 2C), and the non-diabetic mice from HMOS showed higher levels than those from Control group (p < 0.05). In addition, significantly decreased Leptin levels were detected in HMOS-treated NOD-mice as compared to control (p < 0.01, Fig. 2D), and the control diabetic subgroup showed higher levels than all the other three subgroups (p < 0.05, p < 0.05, p < 0.05, Control-Dia vs Control-Non-dia, HMOS-Dia, or HMOS-Non-dia, respectively).

### Dietary HMOS altered the fecal and ceacal microbiota composition of NOD-mice overtime

**Figure 3:**
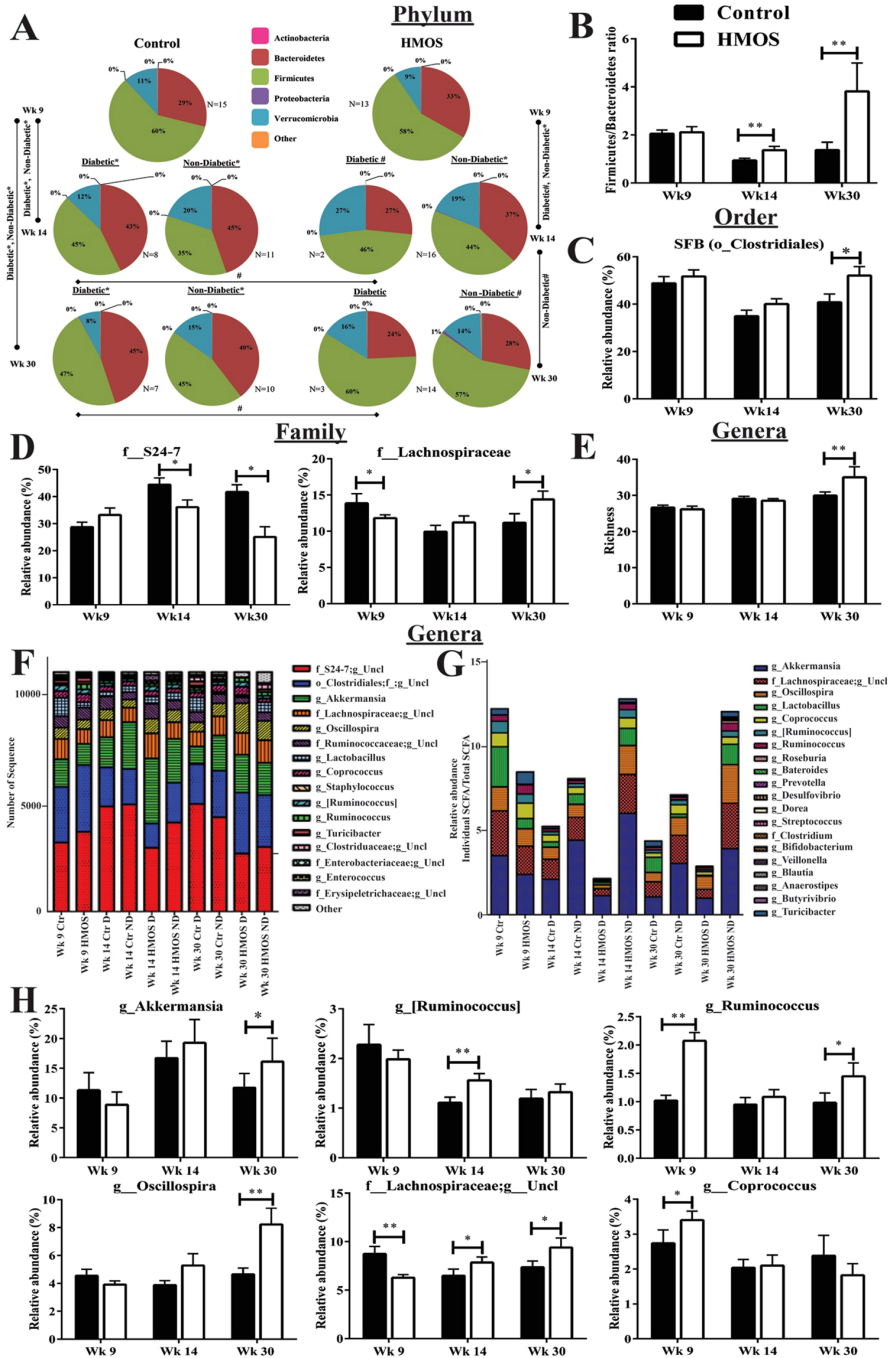
Early HMOS dietary intervention alters fecal microbiota composition over time. Fecal samples at Wk4 (baseline), Wk9 (start intervention), Wk14 (post-intervention) and Wk30 (endpoint) were analyzed using 16 S rRNA. (**A**) Phylum level pie charts, organized by weekly collection time points, significance indicated either by: (^#^)False Discovery Rate (FDR) p < 0.05; (*)Kruskal-Wallis p < 0.05. (**B**) The Firmicutes-to-Bacteroidetes ratio significant differences noted in the fecal microbiota of NOD-mice receiving HMOS or control diet. (**C**) Relative abundance of order level's overall Segmented Filamentous Bacteria (SFB). (**D**) Relative abundance of families S24–7 and Lachnospiraceae in NOD-mice receiving HMOS or control diet. (**E**) The number of observed genera (richness) of bacteria of NOD-mice receiving HMOS dietary intervention or not. (**F**) Stacked column plots of the total number of rarefied sequences (11,000) of the top 90% genera. (**G**) Stacked column plots of relative abundance of genus SCFA-producing bacteria. (**H**) Relative abundance of six different bacterial genera from control and HMOS groups. Data are presented as mean ± SEM, n = 17–20/ group. Statistical differences between groups are depicted as *p < 0.05 and **p < 0.01, using Mann-Whitney U-test.

Knowing that immune and microbiome development are correlated, we analyzed fecal microbiota compositions of NOD-mice receiving either HMOS or control diet using high-throughput 16S ribosomal RNA gene amplicon sequencing at different time points during the experiment. The fecal microbiota of both NOD-mice groups was dominated by three bacterial relative abundance (RA) phyla: Firmicutes, Bacteroidetes, and Verrucomicrobia (Fig. 3A). At baseline (Wk4), no microbial differences were detected prior to intervention (data not shown). Due to HMOS intervention, a gradual change between Firmicutes and Bacteroidetes was detected, leading to a significant increase in the RA of Firmicutes and decrease in the RA of Bacteroidetes at Wk30 (Fig. 3A). Additionally, the Firmicutes-to-Bacteroidetes (F/B) ratio, which has been negatively correlated with glycemic level and reduces overtime in T1D development^17,18^, indeed declined in both groups at Wk9 and Wk14. At Wk14 and Wk30, HMOS-treated NOD-mice showed significantly higher F/B ratio than the control NOD-mice (Fig. 3B). Further analysis between diabetic and non-diabetic NOD-mice, of both control and HMOS-treated groups, indicated significantly higher F/B ratio in the non-diabetic mice at Wk30 (Fig. S3). This data suggest, that HMOS intervention promoted a microbiota composition preceding prevention of T1D development and differed from untreated control NOD-mice.

Segmented filamentous bacteria (SFB) have been positively linked with diabetes protection in NOD-mice^19^. HMOS-treated NOD-mice indicated higher RA of SFB Clostridiales compared to control NOD-mice (Fig. 3C), which lead to a significant difference at Wk30 (p < 0.05). At the taxonomic family level, the RA of S24–7, a dominate bacteria originating from the phylum Bacteroidetes^20^, was significantly reduced in the HMOS-treated NOD-mice at Wk14 and Wk30; whereas the RA of Lachnospiraceae, a member of *Clostridium cluster XIVa* that produces butyrate^21^, was significantly higher in HMOS-treated NOD-mice, when compared to the control mice at Wk30 (Fig. 3D). Additionally, bacterial richness was significantly higher in HMOS-treated NOD-mice compared with control NOD-mice at Wk30 (p < 0.01, Fig. 3E).

The longitudinal study model of RA genera taxa that indicated significance between untreated control and HMOS-treated NOD-mice fecal samples are depicted in (Fig. 3F). Interestingly, we detected increased RA of SCFAs-producing bacteria in HMOS non-diabetic mice (13% at Wk14, and 12% at Wk30) when compared to HMOS diabetic mice (2% at Wk14, and 2.5% at Wk30) (Fig. 3G). Overall, diabetic mice showed reduced SCFA-producer taxa compared to non-diabetic mice at Wk14 and Wk30 in both groups of NOD-mice. It is noteworthy that within those genera, HMOS-treated NOD-mice displayed more abundant *Akkermansia* at Wk30, which is a mucin-degrader in the gut^22^. Besides, the RA of [*Ruminococcus*] (Wk14), *Ruminococcus* (Wk9 and Wk30), *Oscillospira* (Wk30), unclassified genus of *Lachnospiraceae* (Wk14 and 30), and *Coprococcus* (Wk9) were observed significantly higher in HMOS treated mice than in control mice (Fig. 3H).

### Dietary HMOS maintained alpha and beta diversity of the fecal microbiota in NOD-mice

**Figure 4:**
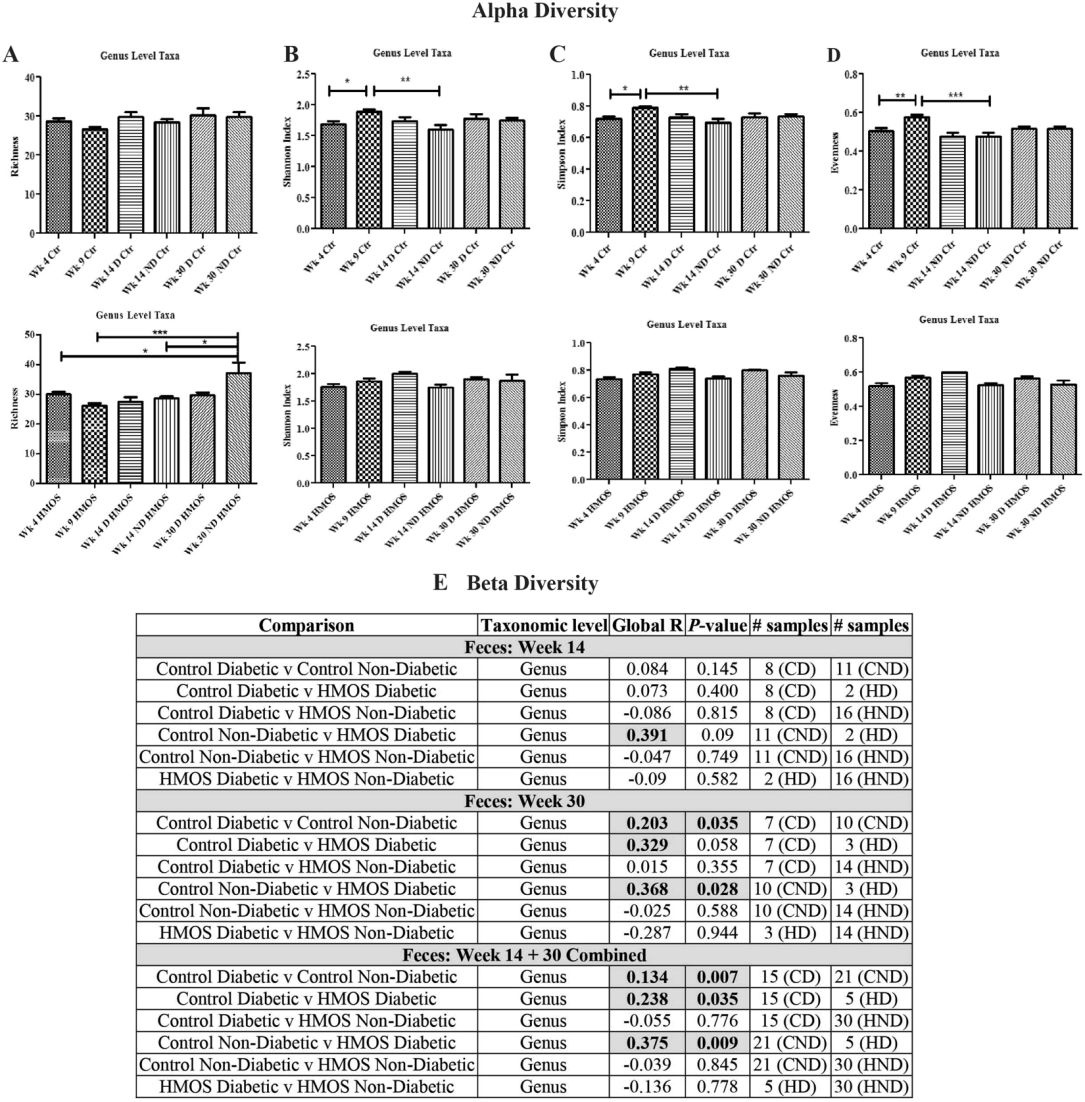
Early HMOS dietary intervention changes the alpha diversity and beta diversity of fecal microbiota of the non-diabetic and diabetic mice within each group at four collection points. (**A**) richness. (**B**) Shannon’s index. (**C**) Simpson’s index. (**D**) evenness. Alpha diversity indices data are represented as mean ± SEM, n = 17–20/ group, *p < 0.05, **p < 0.01, ***p < 0.001, using one-way ANOVA test. (**E**) Inter-group analysis of similarity (ANOSIM) (Beta diversity). Global R comparison was based on ANOSIM performed within the software R package, as described in the text. *P*-values were calculated based on a permutational analysis, employing 9,999 permutations. *p* < *0*.*05*, n = 17–20/group. CD: Control Diabetic; CND: Control Non-diabetic; HD: HMOS Diabetic; HND: HMOS Non-diabetic.

Low diversity of gut microbiota, i.e. number, abundance, and distribution of bacteria has been linked to increased risk of T1D^18,23^. Therefore, we analyzed alpha and beta diversity of fecal microbiota in NOD-mice at different time points. At taxonomic level of genus, no significant changes in alpha diversity indices^24^ were detected in HMOS-treated NOD-mice overtime (Fig. 4B–D), except for bacterial richness (ANOVA; p < 0.01) (Fig. 4A). Particularly, within the HMOS-treated group, significantly higher bacterial richness at Wk30 was detected in non-diabetic mice than in the other disease and time comparisons (Fig. 4A). Additionally, bacterial richness was significantly higher in HMOS-treated NOD-mice compared with control NOD-mice at Wk30 (p < 0.01, Fig. 3E). In contrast, significant loss of diversity in the Shannon (ANOVA; p < 0.01), Simpson (Kruskal-Wallis; p < 0.01), and evenness (ANOVA; p < 0.001) indices were detected in control NOD-mice (Fig. 4B–D). Finally, Wk14 and Wk30 fecal taxon-specific differences were observed within the context of overall microbial community analyses (ANOSIM) that revealed significant differences between the diabetic and non-diabetic NOD-mice in both groups, at the genus level (Fig. 4E). Our finding that NOD mice lose their bacterial diversity over time in T1D development, are consistent with previous studies demonstrating that the level of bacterial diversity diminished overtime in diabetic children^18^ and HMOS dietary intervention seems to prevent NOD-mice from losing their bacterial diversity. This observation was supported by the Bray-Curtis dissimilarity index indicating greater variability in microbial community HMOS non-diabetic mice at Wk30. (Fig. S4).

In addition, functional predictions using PICRUSt analysis indicated that the RA of a majority of reference gene pathways trended different in fecal samples collected from HMOS-treated and control NOD-mice (data not shown). Of note, the RA of three pathways trended higher in HMOS-treated non-diabetic compared to control non-diabetic mice, at Wk14 and Wk30, specifically the carbohydrate digestion and absorption pathway which is associated to SCFAs-producing bacteria (Fig. S5). Overall results indicate and point to specific microbial changes, as well as diversity playing a role in protection of T1D in the NOD-mice receiving early HMOS diet.

**Figure 5:**
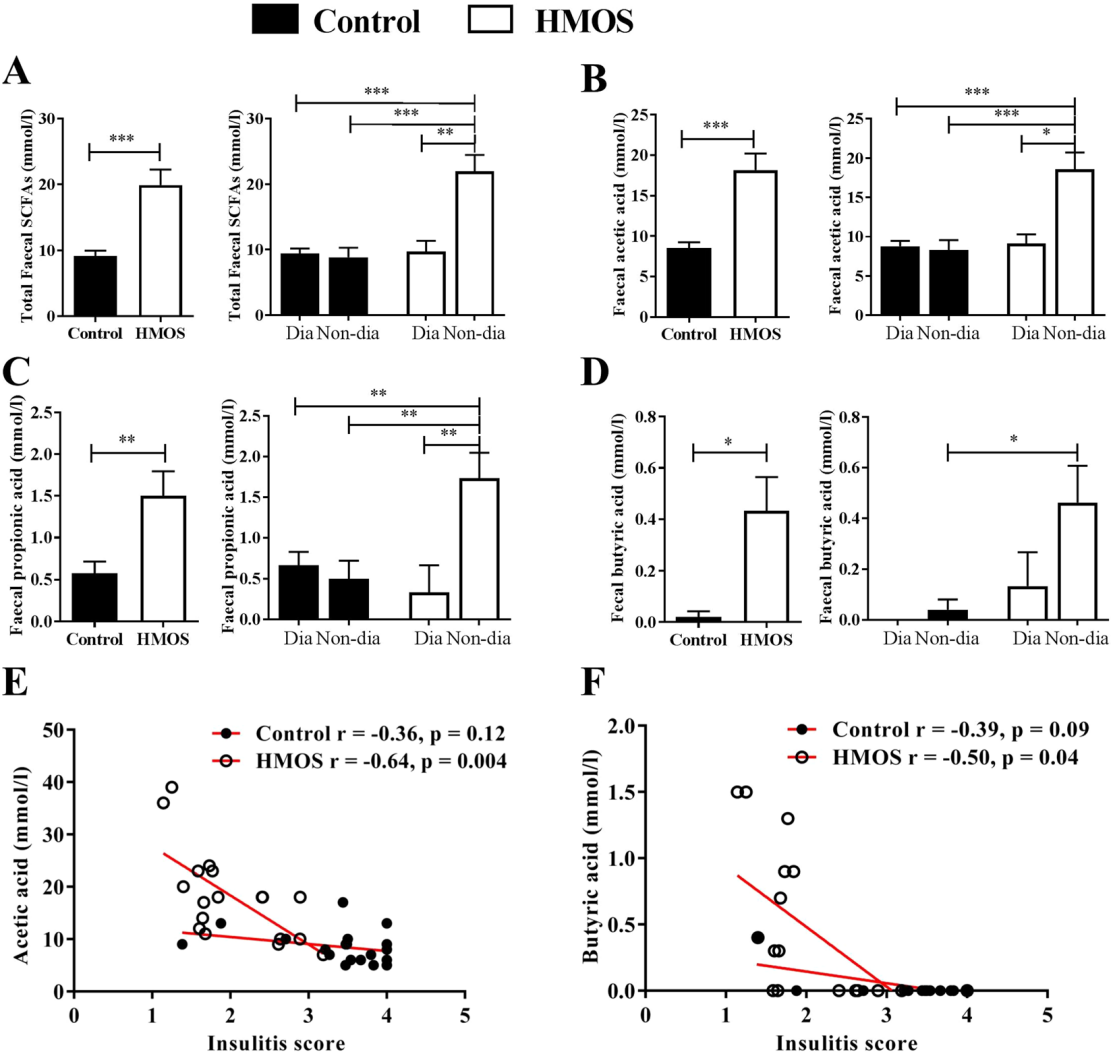
Early HMOS dietary intervention increases production of SCFAs in the fecal samples which are correlated to severity of insulitis. (**A**) Concentrations of total SCFAs (sum of acetic acid, propionic acid, and butyric acid), (**B**) acetic acid, (**C**) propionic acid, and (**D**) butyric acid shown as in total or diabetic (Dia) and Non-diabetic (Non-dia) of control and HMOS group, fecal samples were collected from Wk9. Data are presented as mean ± SEM, n = 17–20/ group. Statistical differences between groups are depicted as *p < 0.05 and **p < 0.01using Mann-Whitney U-test. (**E**) and (**F**) Significant correlations between acetic acid and butyric acid with insulitis score, Spearman correlation analysis was used, *r*- and *p*-value included respectively.

### Dietary HMOS increased fecal SCFAs levels in NOD-mice during dietary intervention

Changes in microbial ecology promoted us to assess SCFAs levels in feces and cecum content by NMR spectroscopy. Within fecal samples from HMOS-treated NOD-mice, a significantly increased total fecal SCFAs (p < 0.0001) concentration was detected compared to control group (at Wk9, which is during the HMOS provision) (Fig. 5A). Specifically, acetic acid (p < 0.0001), propionic acid (p < 0.01), and butyric acid (p < 0.05) were all significantly higher in fecal samples of NOD-mice receiving HMOS− compared to control diets. More importantly, we found that all samples with higher levels of SCFAs were predominantly present in the HMOS-receiving-non-diabetic mice (Fig. 5A–D). In addition, HMOS-treated NOD-mice had relatively higher total SCFAs concentrations detected in cecum content as compared to control mice (Fig. S6). No differences in SCFAs were detected between the groups at Wk14 and Wk30, which are 4 to 20 weeks post HMOS intervention respectively. Consistently with previous study in which SCFAs (specifically, acetate and butyrate) have been demonstrated to directly protect against pancreatic islet inflammation^7^ and diabetes incidence^8^, our correlation analysis between individual SCFAs and insulitis score showed a significantly inverse correlation of acetic acid (spearman correlation R-0.64 and p < 0.01 for HMOS, and R-0.36 with p = 0.12 for control group) (Fig. 5B) and butyric acid (R-0.50 and p < 0.05 for HMOS, and R-0.39 and p = 0.09 for control group) (Fig. 5C) with individual insulitis scores. Together, these results indicate that HMOS mediated protection against development of autoimmune T1D might be established through interaction between microbial derived metabolites SCFA (acetate, propionate, and butyrate) post intervention and immune development.

**Figure 6:**
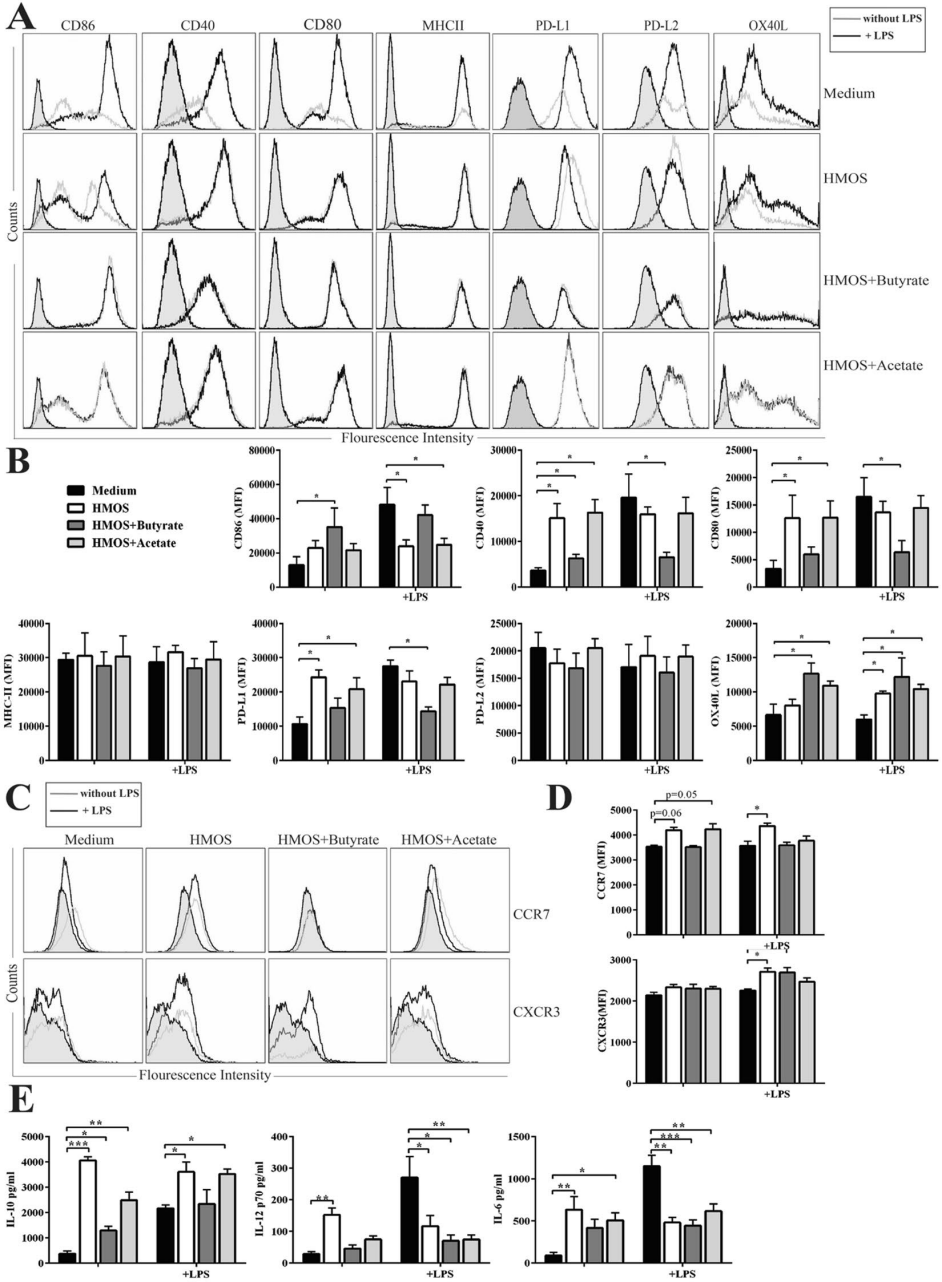
HMOS, HMOS + Butyrate, and HMOS + Acetate induce direct immunomodulatory effects on murine bone marrow derived DCs phenotype and cytokines microenvironment *in vitro*. (**A**) Representative histograms of maturation, co-stimulatory, and inhibitory markers expression on DCs and (**B**) MFI of given markers expression were shown. (**C**) Representative histograms of migratory markers expression on DCs and (**D**) median florescence of intensity of given migratory markers were shown. DCs were treated by medium, HMOS, HMOS + Butyrate, or HMOS + Acetate with (black lines) or without (grey lines) LPS activation for 24 h. Filled histograms represent isotype controlled mAb staining. Gating strategy shown in Fig. S7. (**E**) Concentrations of IL-10, IL-12p70, and IL-6 by medium- (black bars), HMOS− (white bars), HMOS + Butyrate- (dark grey bars), HMOS + Acetate-DC (grey bars. Data are presented as mean ± SEM, n = 3–4 for DCs activation experiment. Statistical differences between groups are depicted as *p < 0.05, **p < 0.01 and ***p < 0.01 using Mann-Whitney U-test.

### HMOS and SCFAs induce tolerogenic DC phenotype *in vitro*

Intestinal produced SCFAs are known to exert their effects in different organs^25^, indeed both acetate and butyrate protect NOD-mice from developing T1D via limiting the autoimmune T cells and promoting functional regulatory T cells^8^. Moreover, specific HMOS, such as 2′-fucosyllactose (2′-FL), 3′-sialyllactose (3′-SL), 6′-sialyllactose (6′-SL), and Lacto-N-neotetraose (LNnT) have been detected within systemic circulation^26,27^, which allows direct interactions with DCs, the pivotal immune regulators that can drive T-cell priming and differentiation. A subpopulation of DCs with tolerogenic phenotypes and functions are suggested to control effector and regulatory mechanisms relevant to pathology of autoimmune diseases such as T1D^28^. Both SCFAs and synthetic oligosaccharides can induce DCs with regulatory phenotypes via distinct mechanisms^29,30^. Therefore, HMOS alone and/or HMOS combined with butyrate or acetate were tested on murine bone marrow derived immature DCs (iDCs) for 24 h after which Th1-type of DCs were induced by adding LPS, DC-phenotype and T-cell induction capacity were subsequently analyzed using flow cytometry. Exposure of iDCs to physiological concentrations of HMOS, HMOS + butyrate, or HMOS + acetate induced a semi-mature phenotype compared to untreated control characterized by induction (MFI) of MHC-II and co-stimulatory molecules CD86, CD80, and CD40 expression, while increased expression of inhibitory molecules PD-L1 and OX40-L were detected (Fig. 6A,B). LPS alone enhanced MFI of MHCII, CD86, CD80, and CD40 on iDCs, but addition of LPS did not change HMOS, HMOS + butyrate, and HMOS + acetate modulated DC-phenotypes (Fig. 6A,B). Interestingly, the expression levels (MFI) of PD-L1 and OX40-L on HMOS− and HMOS + acetate-DC were significantly increased and stable in the presence of LPS as compared to untreated iDCs, pointing to the potential of inducing Tregs^31^ and skewing immune responses^32^. It is worth mentioning that, the observed effects of HMOS are not due to the potential LPS contamination since elimination of LPS by polymyxin B, which is an effective LPS inhibitor, does not influence the HMOS effects (Fig. S8).

CC-chemokine receptor 7 (CCR7) essentially contributes to immunity by guiding cells to and within lymphoid organs^33^. HMOS− and HMOS + acetate-DCs expressed higher levels of CCR7 compared with untreated DCs; LPS up-regulated CCR7 expression in HMOS-DCs. The expression of CXCR3 could only be detected on HMOS-DCs; LPS increased CXCR3 expression on all the three types of DCs (Fig. 6C,D), suggesting increased potential DCs to reach the inflammatory site in the pancreas^33^. This correlated to significant increased release of IL-10 by HMOS−, HMOS + butyrate- and HMOS + acetate-DC compared to untreated DCs, which were reduced upon LPS stimulation. Moreover, IL-12p70, and IL-6 release of HMOS, HMOS + butyrate, and HMOS + acetate treated DCs in the presence of LPS was significantly lower than control LPS-DCs (Fig. 6E), collectively showing a direct modulation of DCs by HMOS and SCFAs.

### HMOS and SCFAs modulate BMDC functions *in vitro*

**Figure 7:**
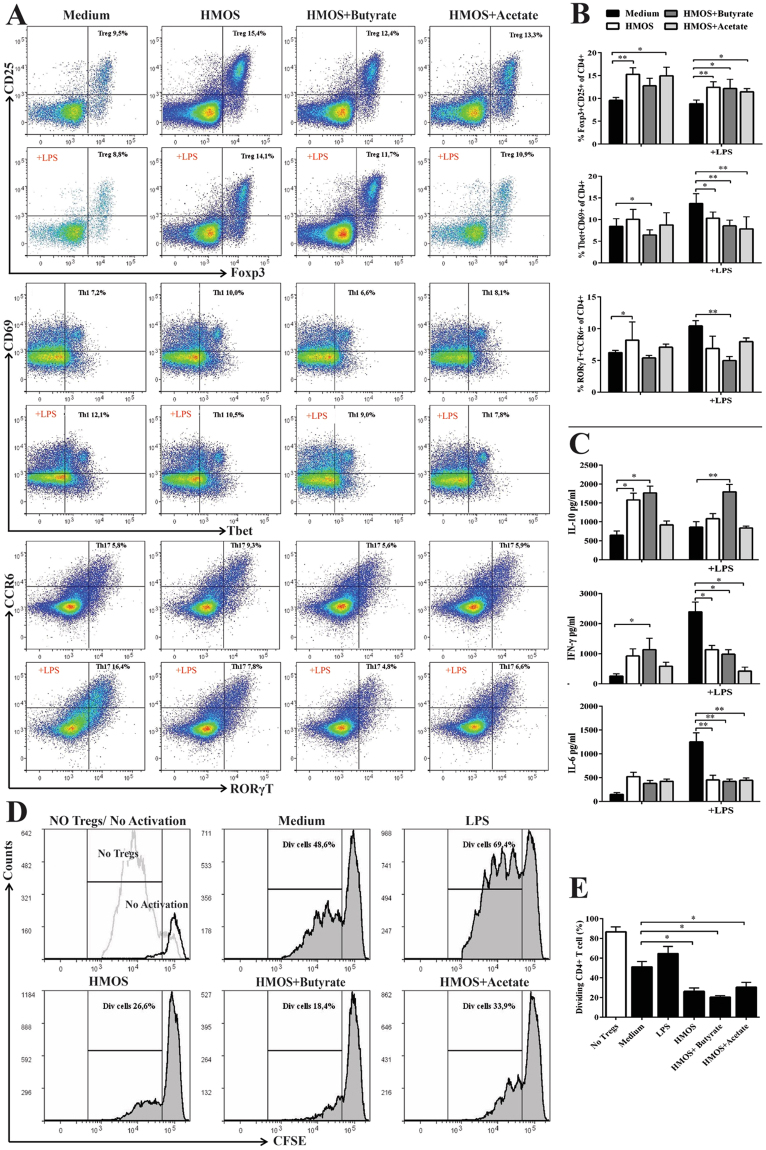
HMOS−, HMOS + butyrate-, and HMOS + acetate-modulated DCs limit effector T cells and promote functional Tregs differentiation. (**A**) Co-cultured splenic naïve T cells were analyzed by flow cytometry, gating strategy shown in Fig. S9. Representative plots of Foxp3 + CD25 + (Treg), Tbet + CD69 + (Th1), and RORγT + CCR6 + (Th17) CD4 T cells primed by different DCs are shown. (**B**) Percentage of Treg, Th1, and Th17 cells of CD4 cells primed by medium- (black bars), HMOS− (white bars), HMOS + butyrate- (dark grey bars), HMOS + Acetate-DC (grey bars). (**C**) IL-10, IFN-γ, and IL-6 levels in the supernatant at day 6 from co-culture. (**D**) Representative plots and (**E**) the degree of responder T cell proliferation after co-culturing with Tregs primed by different DCs, gating strategy is shown in Fig. S10. CFSE-labeled naïve CD4 + T cells as responder cells were co-cultured with Tregs primed by different DCs at ratio of 1:1 for 4 days, CD3/CD28 beads were used to activate the responder T cells. Proliferation of FITC-positive cells was analyzed by flow cytometry and suppressive functionality was determined by comparing the dividing (Div) CD4 + T cells. Data are presented as mean ± SEM, 4 independent allogenic stimulation assays and 3 independent suppressive assays were performed. Statistical differences between groups are depicted as *p < 0.05, **p < 0.01 and ***p < 0.001 using Mann-Whitney U-test.

Knowing that HMOS−, HMOS + acetate-, and HMOS + butyrate modulated DC phenotype, we assessed effect on effector CD4 + T cells activation (i.e Th-1 and Th17-cells) and Treg differentiation. Upon co-culture of HMOS− and HMOS + acetate-modulated DCs with purified naïve splenic CD4 + T-cells significantly higher percentages of Tregs were detected than when co-cultured with untreated DCs (p < 0.01, p < 0.05, respectively), whilst HMOS + butyrate-DC did not change proportion of Tregs compared with untreated iDC (Fig. 7A,B).

In addition, a significant increase of Tbet + CD69 + population (Th1) was induced by HMOS modulated DCs compared with untreated DCs (p < 0.05) (Fig. 7A,B). Upon LPS stimulation, all these three types of DCs, particularly HMOS + acetate-modulated DCs (p < 0.01), significantly decreased the induction of Th1-cells. Unexpectedly, HMOS-DC primed significantly higher percentage of RORγT + CCR6 + (Th17) cells compared with untreated DCs (p < 0.05), whereas DCs stimulated by addition of HMOS and butyrate exhibited relative lower percentage of Th17 cells than untreated DCs (Fig. 7A,B). The addition of LPS in the iDCs was effective in the induction of Th17-cells, however, in the presence of HMOS + butyrate, this effect was significantly reduced (p < 0.01, Fig. 7A,B), which is in line with the reduced cytokine responses (IL-17) detected *in vivo*. The T-cells priming by different DCs was further supported by their cytokine profiles (Fig. 7C). Splenic naïve CD4 + T cells co-cultured with HMOS, and HMOS + butyrate modulated DCs released significantly higher levels of IL-10 compared with those co-cultured with untreated iDCs (p < 0.05). Besides, LPS stimulated DCs induced higher secretion of IFN-γ and IL-6 by the co-cultured cells, and HMOS, HMOS + butyrate, and HMOS + acetate modulated DCs significantly dampened the release of IFN-γ (p < 0.05, p < 0.05, p < 0.05, respectively), and IL-6 (p < 0.01, p < 0.01, p < 0.01, respectively) upon LPS stimulation (Fig. 7C). More importantly, all the Tregs primed by HMOS−, HMOS + butyrate-, HMOS + acetate-modulated DCs displayed a significant higher suppressive capacity in effector T cells compared with untreated iDCs (p < 0.05, p < 0.05, p < 0.05, Fig. 7D,E). These data collectively support that the induction of tDCs by HMOS and microbial derived metabolites may be involved in the prevention of diabetes as observed in NOD-mice receiving HMOS.

## Discussion

Infants’ immune developmental challenge is, providing the ability to maintain tolerance towards self and innocent environmental antigens while inducing protective immunity against pathogens. Breastfeeding is inversely correlated with the risk of developing autoimmune T1D^34^, without knowing the exact protective factors. Here we identified the unique complex mixture of HMOS as critical factor within the anti-diabetogenic benefits of human milk.

Protection beyond HMOS intervention might be mediated by beneficially modifying gut microbiota composition and metabolism, thus regulating the intestinal immunological aberrancies^35^ and maintaining gut integrity. The interaction of microbiota composition and immune system in early development of T1D, suggests an important role for nutrition^36^. The attenuated insulitis by HMOS is consistent with microbiota being a regulator of insulitis in NOD-mice^37^. The loss of bacterial diversity and stability is observed in diabetic children and linked to immunological aberrancies^38^. In contrast to the successive declined of F/B ratio as observed during normal T1D development, HMOS dietary intervention clearly reversed this diabetes driven change overtime and displayed significantly higher F/B ratio at Wk14 and Wk30 compared to control diet (Fig. 3B). Moreover, HMOS significantly promoted the prevalence of probiotic-like bacteria such as SFB (order)^19^ and Lachnospiraceae (family)^39^; whereas, a decrease in the prevalence of diabetogenic microbiome, such as S24–7(family), was detected (Fig. 3D). In humans, lack of SCFAs-producing bacteria (particularly butyrate producers) in fecal microbiota has been associated with increased incidence of T1D^18,22^. One of the most striking results of this study is the increased RA of SCFAs producers in the feces of HMOS-treated NOD-mice, i.e. *Akkermansia* (found increased in NOD-mice receiving HMOS) and *Prevotella* are both putative mucin degraders. The presences of those two specific genera have been suggested as useful indicators of improved gut integrity. In time (this is also reflected in the specific genera), selective changes occur in the microbiome composition of mice receiving HMOS, suggesting possible improvement of gut integrity^22^ via optimizing the gut microbiota composition.

Bacterial metabolites (i.e. SCFAs) produced by commensal bacteria induced by HMOS may play a critical role in the protective effect through different modes of action. HMOS-derived SCFA changes may directly shape the pancreatic environment, resulting in less insulitis. In NOD-mice, SCFAs were shown to decrease inflammation and increased regulation, leading to protection against T1D^7^. As in our study, NOD-mice treated with acetate demonstrated a strong inverse correlation between the concentration of acetate and insulitis score^8^. In addition, the presence of luminal SCFAs, mainly butyrate, have been shown to regulate epithelial barrier function via promoting mucin synthesis^40^. Thus, it is tempting to speculate that increased butyrate concentration subsequently promoting mucin synthesis, with corresponding improvement of barrier integrity, eventually leading to protection against T1D. However, HMOS-derived SCFAs may also modulate systemic and mucosal immune system directly. Observed protection is supported by the anti-diabetogenic cytokines profile induced by HMOS: NOD-mice have an immune profile of excessive Th1- and Th17-polarized immune responses, which could promote pancreatic inflammation^41^. our observation that both IL-17 and INF-γ were lowered, while IL-4 was elevated in the serum of non-diabetic mice of HMOS group only suggests that HMOS skewed over activated Th1/Th17 immune responses of NOD-mice toward a more balanced profile. In addition, the increased TNF-α levels in the late stage of disease contribute to reduced pancreatic islet destruction as detected in HMOS-treated NOD-mice^41,42^. Observed changes in Leptin favor pro-inflammatory cell responses and can directly influence development of autoimmune disease mediated by Th1 immunity^43^. Furthermore, the down-regulated serum IL-17 suggest regulation on gut microbiota-mediated Th17 immunity^44^.

Within NOD-mice the development of T1D can be linked to defects in development and/or functions of dendritic cells, which have shown to be critical in eliciting the auto-immune reaction to beta cells within this model^45^ The potential of tDCs in the prevention and treatment of autoimmune diseases through controlling effector and regulatory mechanisms relevant to the pathology of autoimmunity^28,46,47^, prompted us to study the direct immune modulating potential of HMOS. Indeed, both HMOS alone and concomitant administration of HMOS with specific SCFAs induced tolerogenic phenotypes and regulatory cytokine microenvironment of BMDCs. Direct microbiota-independent immune modulatory properties of HMOS, as well as induced tolerance, by HMOS and SCFAs, were demonstrated in accordance with increased percentage of functional Tregs. This direct interaction between DCs and HMOS and/or SCFAs may specifically occur early in life. These data together point to the possible role the complex mixture of different structures within HMOS mixture play in the direct and/or indirect immune regulation in lymphoid organs and/or pancreas *in vivo*, which might be responsible for the observed protective effects against T1D in NOD-mice.

In conclusion, supplementation of a complex mixture of authentic HMOSstructures early in life has been shown to modulate immune responses in later life and can be an example of immunological programming, as well as microbiome colonization imprinting. We provide for the first time direct evidence that the complex mixture of HMOS, derived from human milk, contributes to the protection of human milk against T1D development. It is worth mentioning that HMOS dietary intervention given to the NOD-mice was only for a short period in the early life, it can be speculated that longer dietary intervention might exert better protective effects since longer duration of breastfeeding has been demonstrated to be more protective than shorter period^2,3^. In future studies, attempts should be made to (1) compare the protective effects of long- and short-term HMOS dietary intervention against T1D, (2) investigate how HMOS modulate the immune responsiveness in e.g. pancreas and MLN, (3) and understand the specific complex mixtures of short-chain and long-chain oligosaccharides of HMOS and their role in the protection against the development of autoimmune diseases.

## Methods

### Human Milk Oligosaccharides

Mature human milk samples donated by healthy donors were pooled and the total fraction of HMOS was purified, resulting in authentic mixture of both short-chain as well as long-chain structures (in approx. 9/1 ratio), including fucosylated and /or sialylated, neutral and acidic oligosaccharides. This resulted in a mixture of 84% HMOS and 16% lactose. Figure 2 shows the HPLC spectrum of HMOS used in our study. Separation and analysis were followed the methods as described previously^47^. Briefly, the lactose was separated from complex mixture of HMOS by SBM chromatography with SEC gel (Toyopearl HW40C) as stationary phase. The total fraction of HMOS was analysed by high performance-size exclusion chromatography; columns: 2xTSK PWXL (300 mm × 7.6 mm I.D.) with precolumn (Tosoh Biosep GmbH, Stuttgart, Germany); eluent: 60 °C deionized water; flow rate: 0.4 ml/min. (For details see: Geisser *et al*. 2005)^48^. The complex mixture of human milk oligosaccharides was added to AIN-93M rodent diet at 10 g/Kg.

### Mice

Non-obese diabetic NOD/ShiLtJ female mice (4 weeks old) of specific pathogen free quality were obtained from The Jackson Laboratory, France. They were housed in sterile individually ventilated cages (IVC-cages) and provided with sterile water, bedding and chow. Food intake and body weight of the individual animal (g) were recorded once a week. The dietary intervention was provided between Wk 4 until Wk 10, after which the control diet (AIN-93M) was administered to both groups until week 30. The animals of each experimental group were spread over 5 cages consisting of 4 animals per cage, and all cages of both groups together were placed according to a Latin-Square design.

### Ethic approval

The human milk sample collection, storage and use were approved and carried out according to European guidelines and regulations^48^. All healthy donors (tested negative for Hepatitis and HIV) provided informed consent to use the voluntary provided human milk sample for Research Purpose in accordance with the Declaration of Helsinki. All experimental protocols and procedures were approved by the Ethical Committee of Animal Research of Utrecht University and the Central Commission for Animal use (Utrecht, The Netherlands, DEC NO. 2011.III.05.052.) and carried out in compliance with the principles of good laboratory animal care following the European Directive for the protection of animals used for scientific purposes.

### Urine and blood glucose measurement

Mice were defined diabetic when weekly monitored urine glucose levels were above 300 mg/dL on two consecutive measurements by a Diastix test strip. Glucose levels were confirmed by testing blood glucose >12 mM using the Ascentia Breeze 2 (Bayer) blood glucose test strip by collecting one blood drop (via cheek punch) of the individual positive mice once a week. Results of blood glucose levels in mice are presented as maximal measured levels of blood glucose in diabetic classified mice (mM). The study was stopped at week 30, whereby the animals were sacrificed and the organs harvested for further analysis.

### Histology

To evaluate the impact of HMOS on the development of insulitis, pancreas was fixed in formalin and embedded in paraffin. 5μm sections were cut using a microtome (Leica Microsystems), and three or four sections were randomly chosen and measured for each pancreas. These were then de-parrafinized, stained with Pertex mounting medium and viewed under a light microscope to assess mononuclear cell infiltration. The degree of insulitis in each animal was determined from several non-consecutive slides cut 100μm apart. Instead of scoring islet insulitis as simply present or not, a classification scheme was implemented for a more accurate statistical analysis. Every islet in each section was scored semi-quantitatively as follows: 0 = No Insulitis; 1 = Peri-Insulitis; 2 = Insulitis affecting less than 50% of the islet area; 3 = Insulitis affecting more than 50% of the islet area; 4 = Complete Insulitis. Average of 46 islets per animal was analyzed.

### Cytokine measurement

Serum IL-2, IL-4, IL-6, IFN-γ, and TNF-α levels were assessed by Luminex multiplex bead assays (ProcartaPlex Mouse High Sensitivity 5-plex, eBioscience, The Netherlands) and IL-10, IL-17, and Leptin were assayed by Luminex multiplex bead assays (customized kit with combinations of IL-10, IL-17, Leptin, eBioscience, The Netherlands) according to manufacturer’s instruction.

### Microbiota profiling and bioinformatics analyses

Fecal pellets of NOD-mice collected from Wk4, Wk9, Wk14, and Wk30 were stored at -80 °C and split into two aliquots for analysis of microbiota or SCFAs. Total DNA was extracted from NOD-mice fecal samples (FastDNA bead-beating Spin Kit for Soil, MP Biomedicals, Solon, OH), amplified the V4 variable region of the microbial 16 S rRNA gene (Earth Microbiome Project primer set, adapted for the Illumina platform)^49^, and sequenced on an Illumina MiSeq (2 × 151 bp reads) at Argonne National Laboratory. Negative controls were used with each set of amplifications, which indicated no contamination. The raw sequence data (FASTQ files) were deposited in the National Center for Biotechnology Information (NCBI) Sequence Read Archive (SRA), under the BioProject identifier PRJNA362610.

Forward and reverse reads were merged and were quality trimmed and sequences shorter than 250 bases were discarded. Sequences were screened for chimeras (usearch61 algorithm)^50^, and putative chimeric sequences were removed from the dataset (QIIME v1.8.0)^51^. Each sample sequence set was rarefied to 11,000 sequences^52^ and data were pooled, renamed, and clustered into operational taxonomic units (OTU) at 97% similarity (usearch61 algorithm). Representative sequences from each OTU were extracted and classified using the uclust consensus taxonomy assigner (Greengenes 13_8 reference database). A biological observation matrix (BIOM)^53^ was generated at each taxonomic level (“make OTU table” algorithm) and analyzed and visualized using the software package Primer7^54^. Alpha diversity indices (within-sample) and beta diversity (between-sample) were used to examine changes in microbial community structure between mice group samples. Alpha diversity indices (i.e., Shannon, Simpson, richness, and evenness) were generated using the package ‘vegan’ implemented in the R programming language. To examine differences in community composition between samples, pairwise Bray-Curtis dissimilarity (non-phylogenetic) metric was generated using the Primer7 software package and used to perform analysis of similarity (ANOSIM) calculations; ANOSIM was performed at the taxonomic level of genus, using square-root transformed data.

### PICRUSt

In-silico community functional predictions were performed using PICRUSt (Phylogenetic Investigation of Communities by Reconstruction of Unobserved States)^55^ and significant differences in Kyoto Encyclopedia of Genes and Genomes (KEGG) functional pathway abundances between groups were identified after filtering to remove low abundance pathways (Kruskal-Wallis test, 1% filter threshold)^56^. KEGG pathways were analyzed using the KEGG Mapper pathway search function^57^.

### SCFAs measurement

SCFAs-acetic acid, propionic acid and butyric acid in the fecal samples and cecum content were measured by Gas Chromatography- as described before^58^. Briefly, the exact weight of Fecal pellets or cecum content were determined and 10 × diluted with ice cold PBS (w/v,) homogenized extensively, centrifuged and supernatants were stored at −80 °C. SCFAs were quantitatively determined by a Varian 3800 gas chromatograph (GC) (Varian, Inc., Walnut Creek, U.S.A.) equipped with a flame ionization detector. 0.5 μL of the sample was injected at 80 °C in the column (Stabilwax, 15 m × 0.53 mm, film thickness 1.00 μm, Restek Co., USA) using helium as carrier gas (3.0 psi). Data are expressed in mmol/g of fecal weight.

### Generation and treatment of DCs

Female, 6–10 week-old C57BL/6JOlaHsd surplus mice were provided to our animal facility at the Central Animal Laboratory (GDL), Utrecht University, The Netherlands. Bone marrow cells were isolated and cultured in RPMI 1640 medium (Gibco) supplemented with 10% FBS for 6 days with 10ng/ml GM-CSF (Prosepec, The Netherlands) to obtain immature DC (iDC). HMOS (5 mg/ml), identical to the batch used within the *in vivo* study, butyrate (2 mM) (Sigma-Aldrich, The Netherlands), acetate (2 mM) (Sigma-Aldrich, The Netherlands), propionate (2 mM) (Sigma-Aldrich, The Netherlands), were added in the presence or absence of LPS (100ng/ml) (eBioscience, The Netherlands) in the iDCs and incubated for 24 hours. iDCs treated with medium were used as control. Phenotypes of DCs were identified using flow cytometry.

### Allogenic Stimulation Assay (ASA) of Naïve T cell

Naïve CD4 + T-cells were isolated from spleen using the negative selection kit (Miltenyi Biotec, The Netherlands). A total of 1 × 10^5^ purified naïve T-cells were co-cultured with treated DCs at a 1:10 ratio in a 96-well U-bottom culture plates for 6 days. T-cell subpopulations were then stained with antibodies and identified by flow cytometry, the gating strategy of T cells subpopulations is shown in Fig. S10. Supernatant was collected at day 6.

### Suppressive assay

To assess the suppressive functionality of regulatory T cells primed by different DCs, whole T cells were harvested from the ASA 96-well plate on day 6, and Tregs were isolated using CD4 + CD25 + T-cell isolation kit (Miltenyi Biotec, The Netherlands) and co-incubated with allogenic freshly isolated responder T cells at a ratio of 1:1 for five days in 96-well U-bottom culture plates. Dynabeads® Mouse T-Activator CD3/CD28 (Thermo Fisher, The Netherlands) were added. Responder cells were stained with Cell Trace CFSE dye (Thermo Fisher, The Netherlands) according to the manufacturers’ instructions at a final concentration of 1uM and FITC-positive cells were acquired. On day 5, the mix of ASA cells and CFSE labeled responder CD4 + T cells was stained with CD4 APC (eBioscience, the Netherlands). Suppressive capacity was determined by setting gates on proliferated cells and comparing the percentage of proliferated cells of different conditions. The gating strategy is shown in Fig. S10.

### Antibodies and Flow cytometry

DCs were harvest after 24 h treatment and stained with different antibodies raised against specific surface markers: Anti-CD11C- PerCP-Cy5.5, Anti-CD86-APC, Anti-CD40-FITC, Anti-CD80-PE-Cy7, Anti-MHCII-PE, Anti-CD274 (PD-L1)-PE, Anti-CD273(PD-L2)-FITC, Anti-OX40-L-APC for 30 mins. Co-cultured cells were harvest and then mixed with different antibodies raised against specific surface markers: Anti-CD4-PerCP-Cy5.5 (eBioscience, The Netherlands), anti-CD25-FITC (eBioscience, The Netherlands), anti-CD69-PE (eBioscience, The Netherlands), anti-CCR-6-PE (eBioscience, The Netherlands), for 30 minutes. Staining for intracellular markers were performed according manufacturer’s protocol, (eBioscience, Foxp3 staining set, Bio connect, The Netherlands). Antibodies used for intracellular markers where anti-Foxp3-PE-Cy7 (eBioscience, the Netherlands), anti-RORγt-PE (eBioscience, The Netherlands). Matching Isotype controls were used to minimize the influence of nonspecific binding and proper gate setting. All incubations were performed on ice and protected from light. In total a minimum of 50,000 cells were counted and analyzed using FACS Canto II and FACS Diva software (BD Biosciences, The Netherlands).

### ELISAs

Culture supernatants of DCs at 24 h, and co-culture cells at 72 h were collected for the presence of cytokines using the Elisa kits. Direct *ex vivo* cytokine secretion was analyzed using IFN-γ, IL-6, IL-10, IL-12p70 capture and detection Ab (eBioscience, The Netherlands) according to the manufacturer’s instructions.

### Statistics

Statistical analysis was performed using GraphPad Prism 6 software. Incidence of diabetes is shown as percentage of diabetic animals in the experimental groups and Log-rank test was performed for significant differences. Data represent mean ± SEM. Statistical analysis on histopathological parameters was performed with the nonparametric Mann–Whitney test. Differences between mean insulitis scores were evaluated using a one-way analysis of variance (ANOVA), with post hoc comparison by the (LSD) method. For the microbiota analysis, differences in relative abundance of individual taxa ( > 1% of dataset), between fecal samples, were assessed for significance using the Kruskal-Wallis test controlling for false-discovery rate, implemented within the software package QIIME^51^. In SPSS, independent *t*-test or ANOVA were used to analyze differences for parametric data satisfying test assumptions; Mann-Whitney U (MWU) test or Kruskal-Wallis were used to analyze nonparametric data. PICRUSt analysis significance was accepted at (p < 0.05), but failed when corrected for FDR-*P*.

### Availability of data and material

The raw data of all 16 S rRNA gene sequencing (FASTQ files) were deposited in the National Center for Biotechnology Information (NCBI) Sequence Read Archive (SRA), under the BioProject identifier PRJNA362610.

## Electronic supplementary material

Supplementary figures
