# Supplementary material for: Human milk oligosaccharides protect against the development of autoimmune diabetes in NOD-mice
Source: Sci Rep. 2018 Mar 1;8:3829. doi: 10.1038/s41598-018-22052-y (PMC5832804; doi:10.1038/s41598-018-22052-y)
Supplement: Supplementary file 1 — Supplementary figures [file 41598_2018_22052_MOESM1_ESM.pdf]

Human milk oligosaccharides protect against the development of autoimmune diabetes in NOD-mice

Ling Xiao[1], Belinda van't Land[2,3*], Phillip A. Engen [4], Ankur Naqib [5], Stefan J. Green[5], Angie Nato[1], Thea Leusink-Muis[1], Johan Garssen[1,2], Ali Keshavarzian[1,4,6], Bernd Stahl[2], Gert Folkerts[1]

[1] Utrecht University, Faculty of Science, Department of Pharmaceutical Sciences, Division of Pharmacology, Utrecht, The Netherlands

[2] Nutricia Research, Department of Immunology / Human milk research platform, Utrecht, The Netherlands

[3] University Medical Center Utrecht, The Wilhelmina Children's Hospital, Laboratory of Translational Immunology, Utrecht, The Netherlands.

[4] Department of Internal Medicine, Division of Digestive Diseases and Nutrition, Rush University Medical Center, Chicago, IL USA

[5] DNA Services Facility, Research Resources Center, Department of Biological Sciences, University of Illinois at Chicago, Chicago, IL USA

[6] Department of Pharmacology, Department of Physiology, Rush University Medical Center, Chicago, IL USA

Figure S1. (A) Average body weight of NOD mice fed with HMOS or control diet overtime. Dietary intervention with HMOS does not change the body weight comapred with non-treatment. **(B)** Occurrence of diabetes. Upper table shows the mice from control group and lower table shows the mice from HMOS group. Red highlights represent mice diagnosed as diabetic at specific week, mice were sacrificed once diagnosed as diabetic.

Figure S2. The spectrum of HMOS

Figure S3. Ratio of Firmicutes/Bacteroidetes in diabetic and non-diabetic mice

Figure S4. The Bray Curtis dissimilarity index. Samples from NOD-mice were devided into diabetic and non-diabetic subgroups. There is greater dissimilarity and variability in the microbial community in Week 30 HMOS Non-Diabetic mice. Broad microbial variability at Week 30 HMOS Non-Diabetic mice could indicate that HMOS is not specific to just one taxa, but HMOS could be altering a greater number of taxa in the overall microbial community.

Figure S5. PICRUSt-predicted metagenomes between the ratio of HMOS non-diabetic to Control non-diabetic mice fecal samples. Feces represent different log10 value fold change functional pathways identified by PICRUSt software ($P<0.05$); 3 pathways were higher abundant in HMOS non-diabetic relative to control non-diabetic NOD-mice.

Figure S6. SCFAs production in cecum content.

Figure S7. The gating strategy used to identify expression of surface markers on DCs.

Figure S8. Effect of polymyxin B on HMOS induced surface markers expression **(A)** and IL-10 secretion **(B)**. HMOS contaning medium was pre-incubated with 10ug/ml polymyxin B for 2h before adding to iDCs for 24h. The expression of CD86, MHC-II, PD-L1on DCs and IL-10 secretion on the DCs supernatant were analyzed. No significant difference was observed between HMOS and pre-treated HMOS. The histograms shown were from one representative experiment and cytokines results were from two independent experiments.

Figure S9. The gating strategy used to identify the different CD4+ T-cell subpopulations.

Figure S10. The gating strategy used to determine CD4 T cells proliferation

Figure S1

A

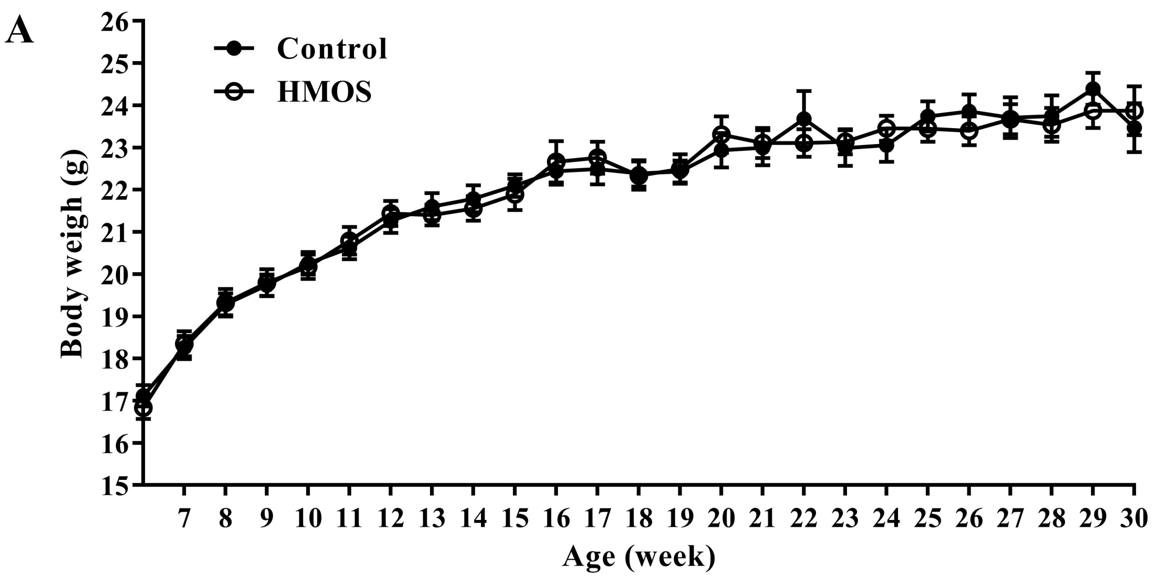

B

1				Age	week 4	week 5	week 6	week 7	week 8	week 9	week 10	week 11	week 12	week 13	week 14	week 15	week 16	week 17	week 18	week 19	week 20	week 21	week 22	week 23	week 24	week 25	week 26	week 27	week 28	week 29	week 30
2	Control	A	1	Urine glucose	0	0	0	0	0	0	0	0	0	0	0	0	0	0	0	4	4										
3	Group	A	2		0	0	0	0	0	0	0	0	0	0	0	0	0	0	0	0	0	0	0	0	0	0	0	0	1	0	0
4		A	3		0	0	0	0	0	0	0	0	0	0	0	0	0	0	0												
5	n=19	A	4		0	0	0	0	0	0	0	0	0	0	0	0	0	0	0	0	0	0	0	0	0	0	0	0	0	0	0
6		A	5		0	0	0		0	0	0	0	0	0	0	0	0	0	4	4											
7		A	6		0	0	0	0	0	0		0	0	0	0	0	0	0	0	0	0	0	0	0	0	0	0	0	0	0	0
8		A	7		0	0	0	0	0	0	0	0	0	0	0	0	0	0	0	0	0	0	0	0	0	0	0	0	0	1	0
9		A	8		0	0	0	0	0	0	0	0	0	0	1	1															
10		A	9		0	0	0	0	0	0	0	0	0	0	0	0	0	0	0	0	0	0	0	0	0	0	0	0	0	0	0
11		A	10		0	0	0	0	0	0	0	0	0	0	0	0	0	0	0	0	0	0	0	0	0	0	1	0	0		
12		A	11		0	0	0	0	0	0	0	0	0	0	0	0	0	0	0	0	0	0	0	0	1	2					
13		A	12		0	0	0	0	0	0	0	0	0	0	0	0	0	0	0	0	0	0	0	0	0	0	0	0	1	0	0
14		A	13		0	0	0	0	0	0	0	0	0	0	0	0	0	0	0	0	0	0	0	0	0	0	0	0	0	4	1
15		A	14		0	0	0	0	0	0	0	0	0	0	0	0	1	0	0												
16		A	15		0	0	0	0	0	0	0	0	0	0	0	0	0	0	0	0	0	0	0	0	0	0	0	0	0	0	0
17		A	16		0	0	0	0	0	0	0	0	0	0	0	0	0	0	0	0	0	0	0	0	0	0	0	0	0	0	0
18		A	17		0	0	0	0	0	0	0	0	0	0	0	0	0	0	0	0	0	0	0	0	0	0	0	0	0	0	0
19		A	18		0	0	0	0	0	0	0	0	0	0	0	0	0	0	0	0	0	0	4	4							
20		A	19		0	0	0	0	0	0	0	0	0	0	0	0	0	0	0	0	0	0	0	0	0	0	0	0	0	0	0
21																															
22				Age	week 4	week 5	week 6	week 7	week 8	week 9	week 10	week 11	week 12	week 13	week 14	week 15	week 16	week 17	week 18	week 19	week 20	week 21	week 22	week 23	week 24	week 25	week 26	week 27	week 28	week 29	week 30
23	HMOS	B	1	Urine glucose	0	0	0	0	0	0	0	0	0	0	0	0	0	0	0	0	0	0	0	0	0	0		0	0	0	0
24	Group	B	2		0	0	0	0	0	0	0	0	0	0	0	0	0	0	0	0	0	0	0	0	0	0	0	0	0	0	0
25		B	3		0	0	0	0	0	0	0	0	0	0	0	0	0	0	0	0	0	0	0	0	0	0	0	0	0	0	0
26	n=20	B	4		0	0	0	0	0	0	0	0	0	0	0	0	0	0	0	0	0	0	0	0	0	0	0	0	0	0	0
27		B	5		0	0	0	0	0	0	0	0	0	0	0	0	0	0	0	0	0	0	0	0	0	0	0	4	4		
28		B	6		0	0	0	0	0	0	0	0	0	0	0	0	0	0	0	0	0	0	0	0	0	0	0	0	0	0	0
29		B	7		0	0	0	0	0	0	0	0	0	0	0	0	0	0	0	0	0	0	0	0	0	0	0	0	1	0	
30		B	8		0	0	0	0	0	0	0	0	0	0	0	0	0	0	0	0	0	0	0	0	0	0	0	0	0	0	0
31		B	9		0	0	0	0	0	0	0	0	0	0	0	0	0	0	0	0	0	0	0	0	0	0	0	0	0	0	0
32		B	10		0	0	0	0	0	0	0	0	0	0	0	0	0	0	0	0	0	0	0	0	0	0	0	0	0	0	0
33		B	11		0	0	0	0	0	0	0	0	0	0	0	0	0	0	0	0	0	0	0	0	0	0	0	0	0	0	0
34		B	12		0	0	0	0	0	0	0	0	0	0	0	0	0	0	0	0	0	0	0	0	0	0	0	0	0	0	0
35		B	13		0	0	0	0	0	0	0	0	0	0	0	0	0	0	0	0	0	0	0	0	0	0	0	0	0	0	0
36		B	14		0	0	0	0	0	0	0	0	0	0	0	0	0	0	0	0	0	0	0	0	0	0	0	0	0	0	0
37		B	15		0	0	0	0	0	0	0	0	0	0	0	0	0	0	0	0	0	0	0	0	0	0	0	0	0	0	0
38		B	16		0	0	0	0	0	0	0	0	0	0	0	0	0	0	0	0	0	0	0	0	0	0	0	0	0	0	0
39		B	17		0	0	0	0	0	0	0	0	0	0	0	0	0	0	0	0	0	0	0	0	0	0	0	0	0	0	0
40		B	18		0	0	0	0	0	0	0	0	0	0	0	0	0	0	0	0	0	0	0	0	0	0	0	0	0	0	0
41		B	19		0	0	0	0	0	0	0	0	0	0	0	0	0	0	0	0	0	0	0	0	0	0	0	0	0	0	0
42		B	20		0	0	0	0	0	0	0	0	0	0	4	4															

Figure S2

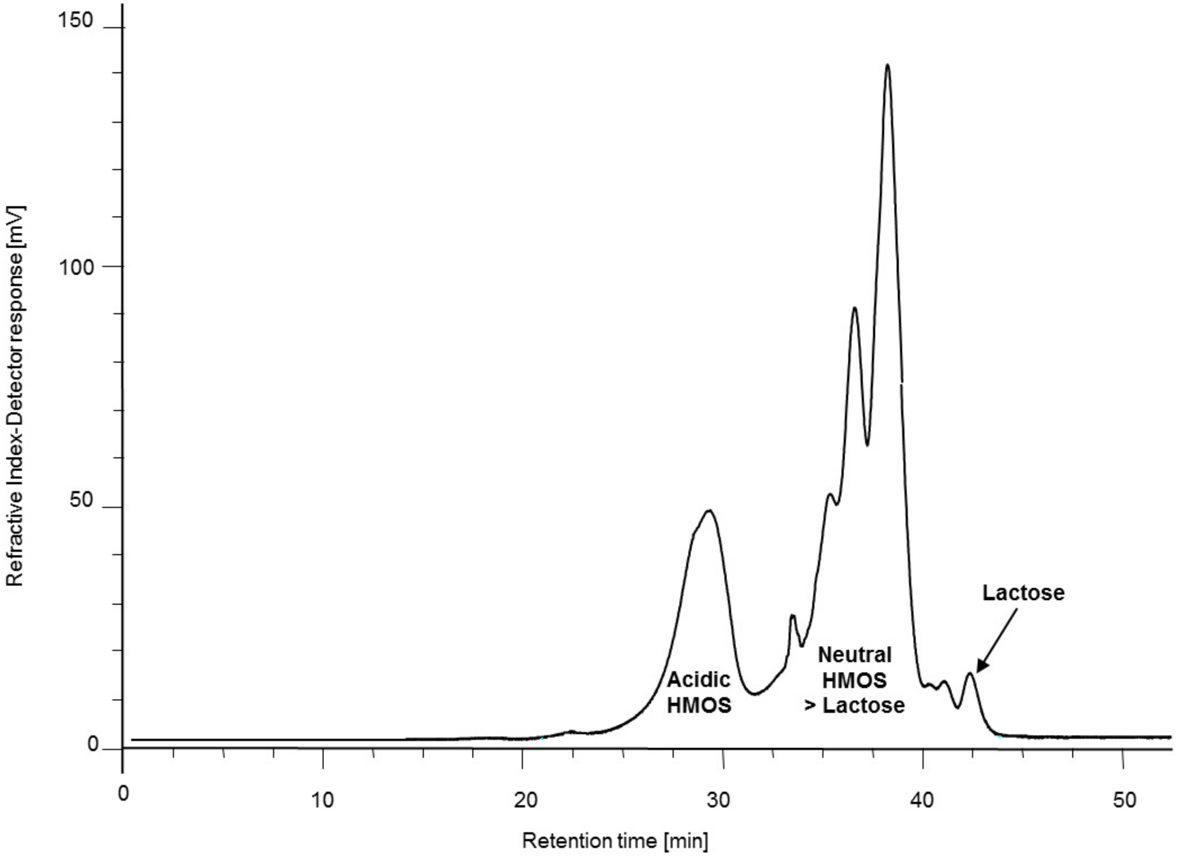

Figure S3

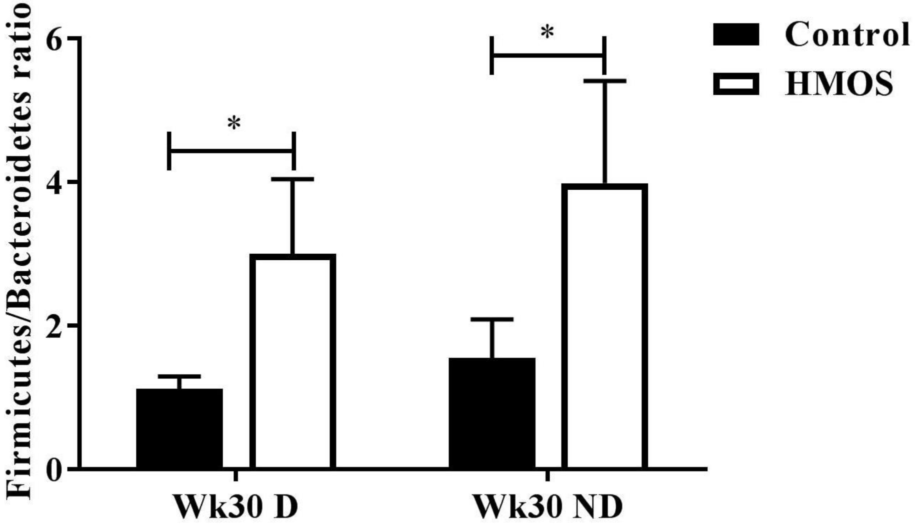

Figure S4

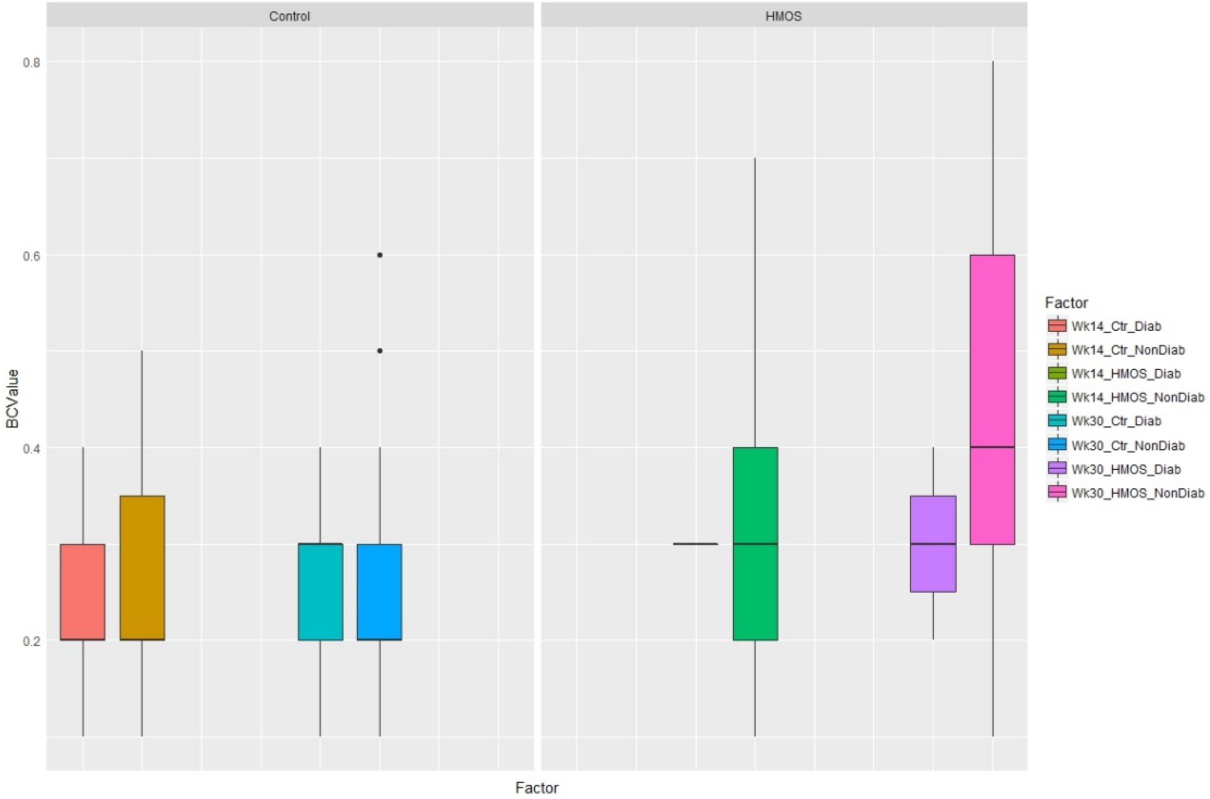

Figure S5

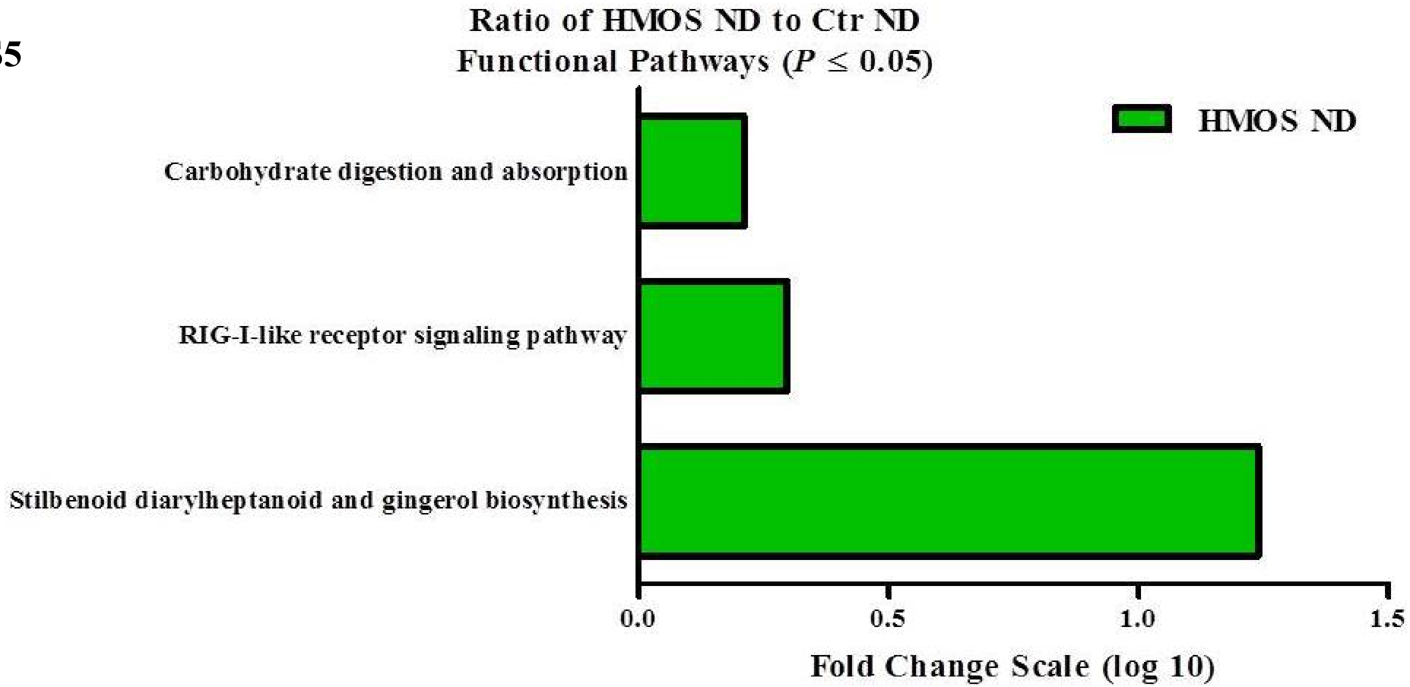

Figure S6

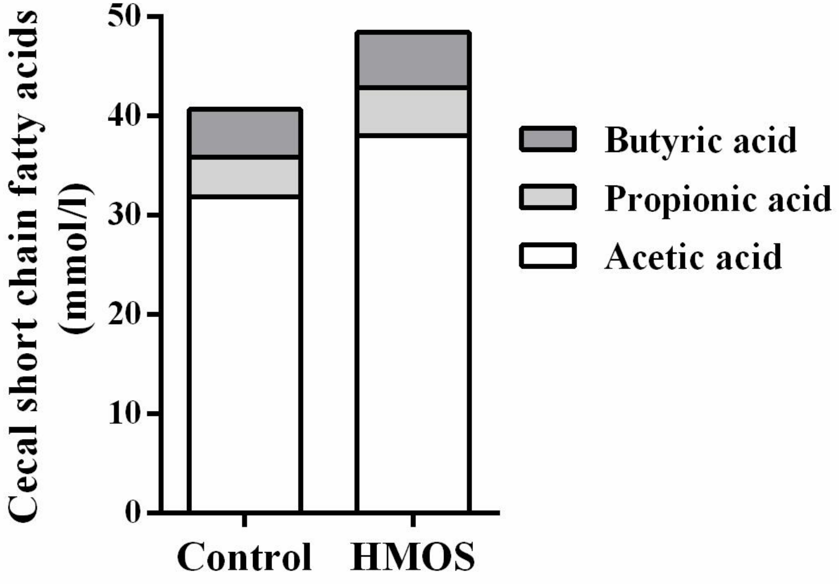

Cecal short chain fatty acids (mmol/l)
50
40
30
20
10
0
Control
HMOS
Butyric acid
Propionic acid
Acetic acid

Figure S7

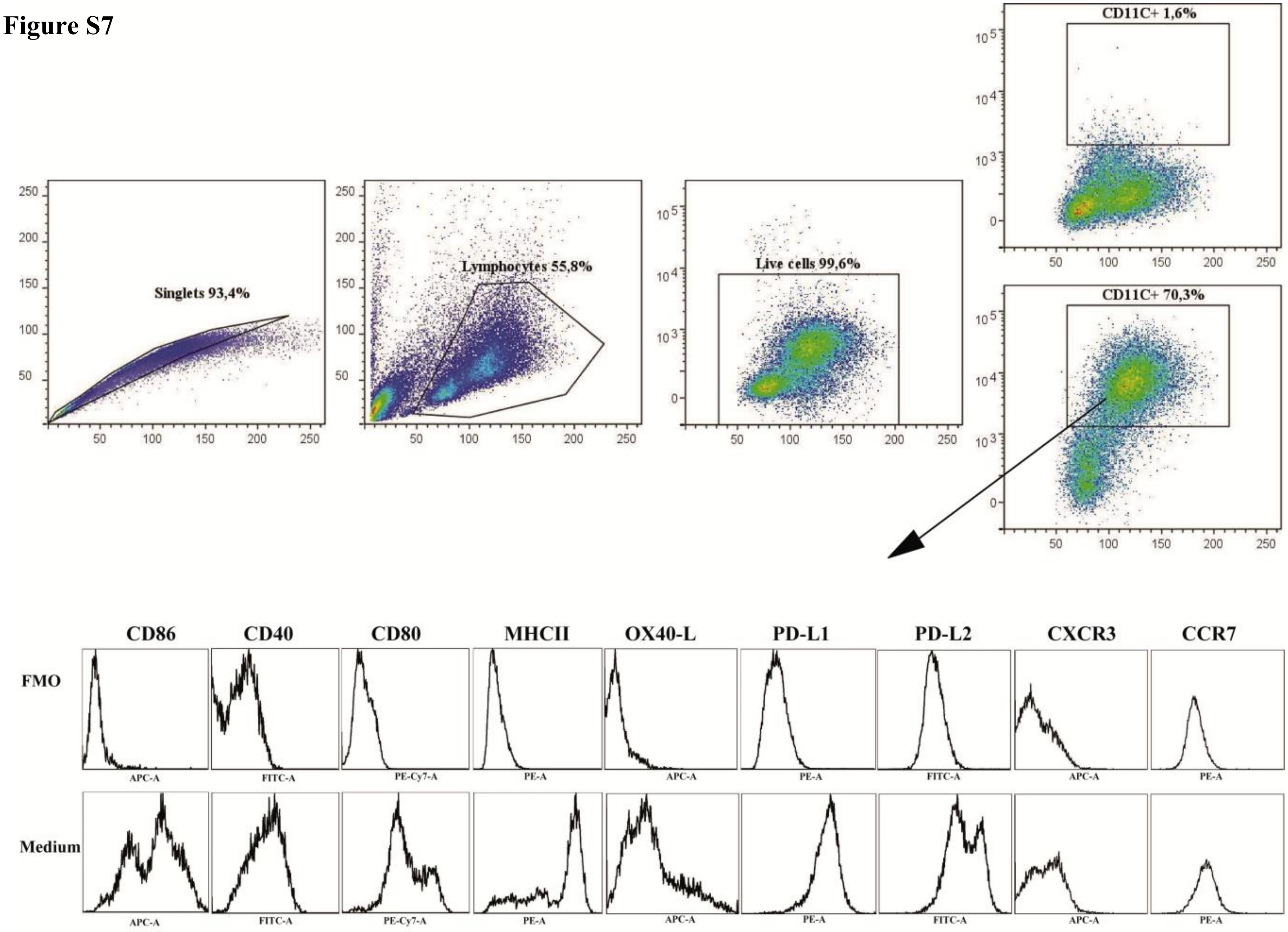

CD11C+ 1,6%
Singlets 93,4%
Lymphocytes 55,8%
Live cells 99,6%
CD11C+ 70,3%
CD86
CD40
CD80
MHCII
OX40-L
PD-L1
PD-L2
CXCR3
CCR7
FMO
Medium
APC-A
FITC-A
PE-Cy7-A
PE-A

Figure S8

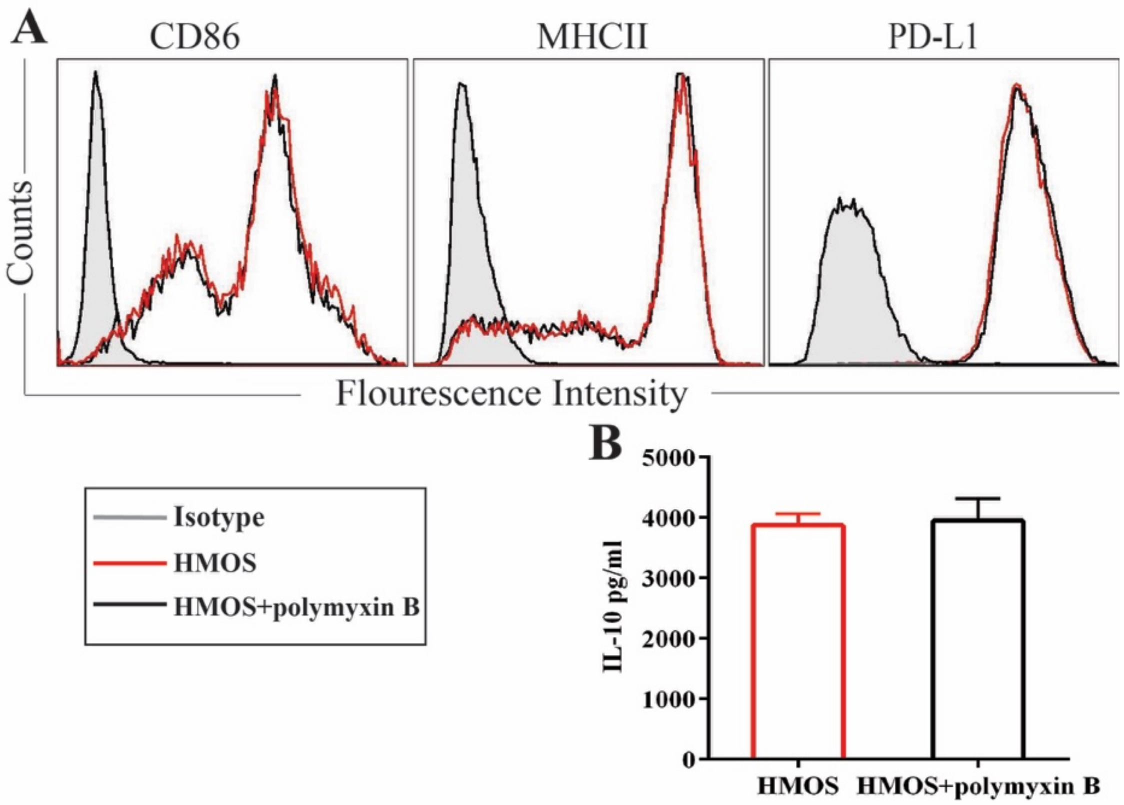
A
CD86
MHCII
PD-L1
Counts
Flourescence Intensity
Isotype
HMOS
HMOS+polymyxin B
B
5000
4000
3000
2000
1000
0
IL-10 pg/ml
HMOS
HMOS+polymyxin B

Figure S9

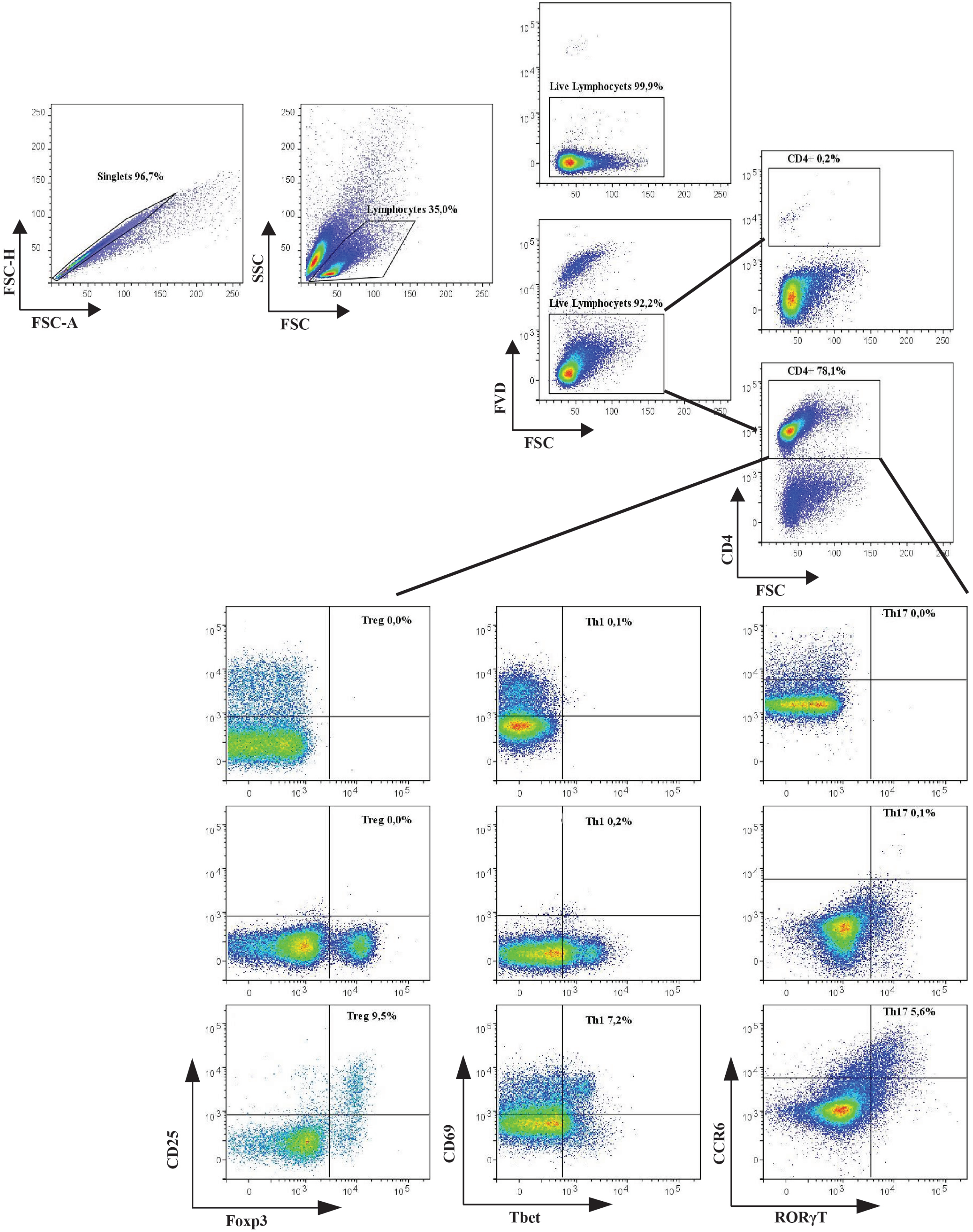

Singlets 96,7%
FSC-H
FSC-A
Lymphocytes 35,0%
SSC
FSC
Live Lymphocyets 99,9%
Live Lymphocyets 92,2%
FVD
FSC
CD4+ 0,2%
CD4+ 78,1%
CD4
FSC
Treg 0,0%
Treg 0,0%
Treg 9,5%
CD25
Foxp3
Th1 0,1%
Th1 0,2%
Th1 7,2%
CD69
Tbet
Th17 0,0%
Th17 0,1%
Th17 5,6%
CCR6
RORγT

Figure S10

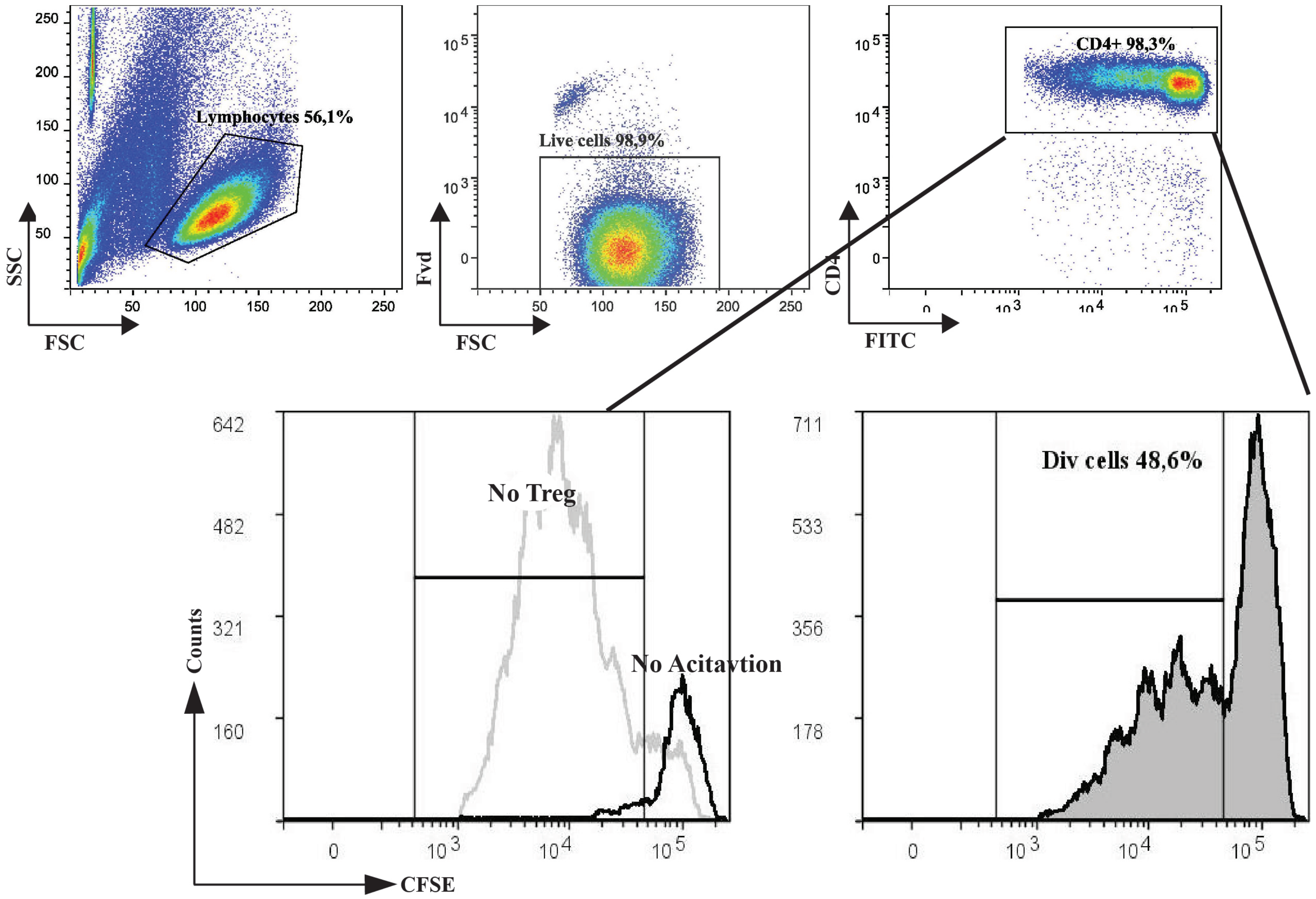

Lymphocytes 56,1%
SSC
FSC
Live cells 98,9%
Fvd
FSC
CD4+ 98,3%
CD4
FITC
No Treg
No Acitavtion
Counts
CFSE
Div cells 48,6%
